# Targeted elimination of molybdenum ions from a leaching solution with the ability of radiated grafting GMA-PAN nanofibers

**DOI:** 10.1038/s41598-023-50608-0

**Published:** 2024-01-02

**Authors:** Mohammad Reza Fayazi, Mohammad Outokesh, Mehdi Asadollahzadeh, Meisam Torab-Mostaedi, Rezvan Torkaman

**Affiliations:** 1 Department of Energy Engineering, Sharif University of Technology, P.O. Box: 11365-8639, Tehran, Iran; 2 Nuclear Fuel Cycle Research School, Nuclear Science and Technology Research Institute, P.O. Box: 11365-8486, Tehran, Iran

**Keywords:** Chemical engineering, Other nanotechnology

## Abstract

In this study, electrospun polyacrylonitrile nanofibers were effectively functionalized for enhanced molybdenum ion adsorption through a multi-step approach. Initially, glycidyl methacrylate was grafted onto the nanofibers via irradiation-induced grafting polymerization, followed by chemical modification with various amino groups, with triethylamine identified as the optimal modifier. The impacts of key synthesis parameters and reaction conditions on grafting level and adsorption capacity were thoroughly investigated, with a focus on achieving maximum efficiency. The resulting nanofibers were characterized using FTIR, SEM, and BET techniques, confirming the successful modification and structural features conducive to adsorption. Furthermore, a comprehensive experimental design, incorporating a central composite design, yielded optimal conditions for molybdenum adsorption, with key parameters including monomer concentration, irradiation dose, adsorbent mass, initial concentration, time, pH, temperature, and amine concentration. The adsorption kinetics were effectively described by the pseudo-second-order model, while the Langmuir isotherm model provided valuable insight into the adsorption behavior. Impressively, the adsorbent exhibited exceptional adsorption efficiency, surpassing 98% even after six adsorption–desorption cycles using 0.5 M HCl. Thermodynamic analysis revealed the exothermic nature of the adsorption process, along with decreased entropy and overall spontaneity, underlining the favorable conditions for molybdenum adsorption. Notably, the synthesized adsorbent demonstrated notable selectivity for molybdenum and achieved an impressive adsorption capacity of 109.79 mg/g, highlighting its potential for practical applications in molybdenum removal from aqueous solutions.

## Introduction

Molybdenum is a transition metal of strategic and industrial importance due to its numerous uses. The main applications are an alloying agent in steels, cast iron, reactor vessels, special batteries to improve hardenability, strength, and corrosion resistance, and chemical applications, such as catalysts, lubricants, and pigments^1^. The high-level waste of the PUREX reprocessing process has a sizable concentration of molybdenum, one of the fission products. Because of molybdenum’s complicated chemical makeup, it may create both soluble and insoluble complexes, which can cause issues with high-level waste concentration, storage, and solidification, among other topics. As a result, molybdenum concentration should be controlled, and its extra should be removed or adsorbed^2^. Although molybdenum may exist in oxidation levels from − 2 to + 6, Mo(VI) is typically considered the dominating oxidation state in polluted natural waters^3^.

In general, various techniques may be used to separate heavy metal ions, including electrolysis, liquid membrane separation, liquid chromatography, ion exchange, liquid–liquid extraction (LLE), adsorption procedure, and chemical precipitation^4^. Adsorption is one of these methods, that has the benefits of effectiveness and cost viability for removing traces from water^5^.

Numerous sorbents have been found to date that may adsorb molybdenum, these sorbents have significant drawbacks, including low selectivity, limited capacity for reuse, and delayed adsorption–desorption kinetics. Thus, it is crucial to develop a new adsorbent with high adsorption selectivity, high recycling potential, and rapid adsorption–desorption rates^6^.

As substrates, a variety of materials in different forms have been used, including membranes^7^, nonwoven fabrics^8^, porous hollow-fiber membranes^9^, fibers^10^, plant fibers^11^, and particles^12^. Due to their large surface area-to-unit mass ratios, nanomaterials have recently attracted a lot of attention in developing adsorbents.

Numerous nano-adsorbents have been studied, including nanobeads^13^, nanocomposites^14^, magnetic-nano adsorbents^15^, and nanofiber mats^16^. Their various benefits include high porosity, high gas permeability, and high specific surface area per unit mass, which should result in a high adsorption capacity. Therefore, nanofiber mats have received a lot of attention.

There are several ways to create nanofibers, including template synthesis^17^, self-assembly^18^, solution blow spinning^19^, drawing spinning^20^, and electrospinning^21,22^. The manufacture of nanofibers using the straightforward and adaptable electrospinning process is well known^23^. A metallic capillary connected polymer solution reservoir with high voltage and a metallic collector are standard components of an electrospinning system. When a high voltage is supplied to a polymer solution, the hemispherical form of the droplet is destabilized by the built-up charges on the surface and changes to Taylor’s cone. A jet of very fine fibers is created from the tip of the Taylor cone when the voltage reaches a critical level when the electric forces override the surface tension on the droplet.

An essential polymer with numerous advantageous characteristics, such as chemical stability, resistance to corrosion and biodegradation, and ease of preparation into nanofiber mats by electrospinning, polyacrylonitrile (PAN) is commonly used to create fiber membranes^24,25^. It has been discovered that an adsorbent containing nitrogen-based functional groups was efficient in the adsorption or removal of heavy metal ions due to the abundance of nitrile groups (C≡N) on the surface of PAN fiber^26^. However, modifications to nanofibers are necessary owing to their low electrospun ability and restricted capacity to bind heavy metal ions^27^.

Numerous functional groups, including carboxylic acid, hydroxyl group, sulfonic acid, and amino group, were added to nanofibers to enhance their capabilities^28^.

An easy method to change and enhance polymeric material is radiation-induced graft polymerization (RIGP). Radiation, plasma, light, and chemicals are examples of excitation sources for the graft polymerization process that produces radicals on polymer substrates. A high density of electron beams or gamma rays may create a lot of radicals in polymers of different components and shapes, radiation-induced graft polymerization is better than other grafting approaches when employing these sources^29,30^. Ibrahim et al. prepared chitosan-graft maleic acid by using gamma radiation to remove copper and nickel ions from aqueous solutions^31^, Saleh et al. employed poly (chitosan-acrylamide) as an adsorbent by using gamma radiation for adsorption of copper(II) and nickel(II) from aqueous solution^32^, Also, Saleh and co-workers reported the grafting of the maleic acid and acrylamide onto chitosan by using gamma radiation for removing Co(II) from aqueous solutions^33^.

Pre-irradiation and simultaneous irradiation graft polymerization techniques are the two categories of radiation-induced graft polymerization^34^. In the first technique, the polymer substrates must be irradiated before a monomer is grafted. Still, in the second way, both polymer substrates and monomers must be irradiated at the same time for radical production and grafting. The substrate's surface is given a homopolymer via the simultaneous irradiation approach. Radicals are produced in both the substrate and the solvent molecules in the solution, which causes the polymer to grow from all of the radicals. As a result, both too much-grafted polymer on the surface and ungrafted polymer in the solution is produced. A three-dimensional crosslinked polymer network and homopolymer are created by the simultaneous irradiation of the grafted and ungrafted polymers. The unwanted homopolymer from this process may be reduced by using an inhibitor salt^35^. Applications for radiation-induced graft polymerization (RIGP) fall into three categories: polymeric membranes, polymeric adsorbents, and graft polymers for use in biotechnology, medicine, and the environment^36–38^.

A typical reactive monomer is glycidyl methacrylate (GMA)^39^. The ability to further modify grafted goods benefits from the graft polymerization of GMA onto polymer surfaces. Because a variety of functional groups, including amino and thiol groups, readily combine with the epoxy group of GMA^40^.

The experimental parameters, such as the component and form of the polymer substrates, the irradiation dosage, the kind of monomer, and the solvents, may impact the quantity of grafting monomer on the polymer substrate^41^.

The primary benefit of an adsorbent for the adsorption of metal ions is their selectivity, which depends on any functional groups present on their surfaces, such as thiol, iminodiacetate, amine, amide, carboxylic acid, hydroxyl, and sulfonic acid^42^.

One of the best chelating functional groups for the adsorption or removal of heavy metal ions from aqueous solutions has been determined to be amino groups, an anion metal ion may be adsorbed by an amino group by electrostatic contact^43,44^.

This work included the radiation-induced grafting of GMA onto polyacrylonitrile (PAN) fiber and subsequent amination with triethylamine (TEA) to create a new chelating fiber containing amino groups. The degree of grafting and adsorption capacity were examined due to the reaction parameters. The adsorption capacity of the grafted adsorbent is improved by the presence of two distinct functional groups in tertiary amine methacrylate, namely carboxyl and tertiary amino groups. The beginning pH, contact duration, adsorbent mass, initial ion concentration, and reaction temperature are typical influences on adsorption behavior that are significant in defining the process's limiting factor^45^.

Different adsorbent synthesis conditions and different reaction conditions, such as monomer concentration, radiation dose, solvents, amine concentration, initial pH of the solution, contact time, adsorbent dosage, initial molybdenum concentration, and temperature, can be studied using a variety of tools, including the statistical and quantitative formulas in Design Expert^46,47^.

One approach for experimental modeling and design is the response surface methodology (RSM)^48,49^. Finding the best inputs, addressing issues, and strengthening the process are some of the objectives of RSM. The program’s findings are then statistically reviewed, and the excellent value for each parameter in this experiment is then determined. The ability to provide a quantitative link between independent and dependent variables is one benefit of employing the response surface methodology and minimizing the number of trials^50,51^.

To determine the maximal effect and improve the test conditions, Mo(VI) was adsorbed from an aqueous solution using the response surface methodology in this work. This method generates the test matrix by using parameters for the number of variables and the maximum and minimum numbers specified for each variable. Because it requires fewer tests than cumbersome techniques like complete factorial, this approach is preferred^52^. The use of radiation for the production of grafted polymeric adsorbent for the adsorption of molybdenum ions has only received little research attention^53^. The current work demonstrated the direct gamma-ray irradiation to glycidyl methacrylate monomer and nanofiber to create a new polymer adsorbent to clarify the influence of critical factors on the adsorption efficiency, the effect of various amines on the alteration of the adsorbent structure. The adsorption studies using the central composite design (CCD) a component of the RSM^54^.

## Experimental

### Materials

The PAN industrial was provided by Taekwang Industry (Korea). Dimethylformamide (DMF; ~ 99.98% purity, Sigma-Aldrich), Glycidyl methacrylate (${\text{C}}_{7} {\text{H}}_{10} {\text{O}}_{3}$,  ≤ 97%, Sigma-Aldrich), methanol (${\text{CH}}_{3} {\text{OH}}$, Merck), ethylenediamine (EDA > 99%, Merck), diethylamine (DEA > 99%, Merck), ethanolamine (EA > 99%, Merk), triethylamine (TEA > 99%, Merck), ferrous sulfate heptahydrate (${\text{FeSO}}_{4} .7{\text{H}}_{2} {\text{O}}$,Sigma-Aldrich), sodium molybdate dihydrate (${\text{Na}}_{2} {\text{MoO}}_{4} .2{\text{H}}_{2} {\text{O}}$; ~ 99% purity, Sigma Aldrich), Deionized water (DI).

### Methods

#### Electrospinning of PAN

Dimethylformamide (DMF) was added to a homogenous electrospinning precursor solution of PAN (15 wt%), and the mixture was stirred by a magnetic stirrer for 24 h. A 5 mL glass syringe was fitted with a needle tip that was 0.5 mm in diameter. A voltage of between 15 and 17 kV was applied to the needle, and 130 mm was the distance between the needle tip and collector. The solvent was evaporated during the electrospinning process at 303 K. A jet of the polymer solution emerged from the needle tip and was gathered on the collector at a critical voltage. PAN nanofiber was collected and electrospun continuously at 1 mL/h for 4 h.

#### Radiation-induced grafting on nanofiber

First, a monomeric solution of GMA was used to submerge the synthesized polyacrylonitrile fibers and to avoid the development of homopolymer reactions, methanol and ferrous sulfate heptahydrate salt (0.5% wt) were utilized. The vial carrying the PAN nanofiber and the monomeric solution was then filled with nitrogen gas and sealed to prevent the reactivity of radicals produced by air oxygen. The produced vial was exposed to gamma radiation using the simultaneous irradiation polymerization technique at dosages ranging from 10 to 50 kGy. The grafted PAN nanofibers were washed in methanol to remove the residual monomer and homopolymer. The GMA grafted onto PAN (GMA-g- PAN) fibers were dried for 24 h at 308 K in a vacuum oven before being weighed. The following formula was used to determine the degree of grafting^55^:

1 $$ D_{g} \left( \% \right) = \frac{{W_{g} - W_{0} }}{{W_{0} }} \times 100 $$

where $W_{0}$ and $W_{g}$ are the weights of the original and grafted PAN fibers, respectively.

#### Amination of grafted polymer

The functionalization of the GMA-g-PAN nanofiber samples was accomplished utilizing four amination agents, including EA (primary amine), EDA (primary amine), DEA (secondary amine), and TEA (tertiary amine), in four different processes. A sample of GMA-g-PAN nanofiber of known weight was used to carry out the reaction and the opening of the epoxy rings found in the GMA-g-PAN side chains with amine led to the amination process. In the amination using EA, EDA, and DEA, a solution of the amine agent was diluted in water to concentrations of 60% (v/v) and the solution was stirred at 333 K for 4 h, in contrast for TEA, the solution was diluted in methanol to concentrations of 60% (v/v). In adsorption studies, it was shown that utilizing TEA for the amination process rendered the samples very brittle and challenging to handle. Therefore, the reaction was run at 303 K for 8 h^56^.

**Figure 1:**
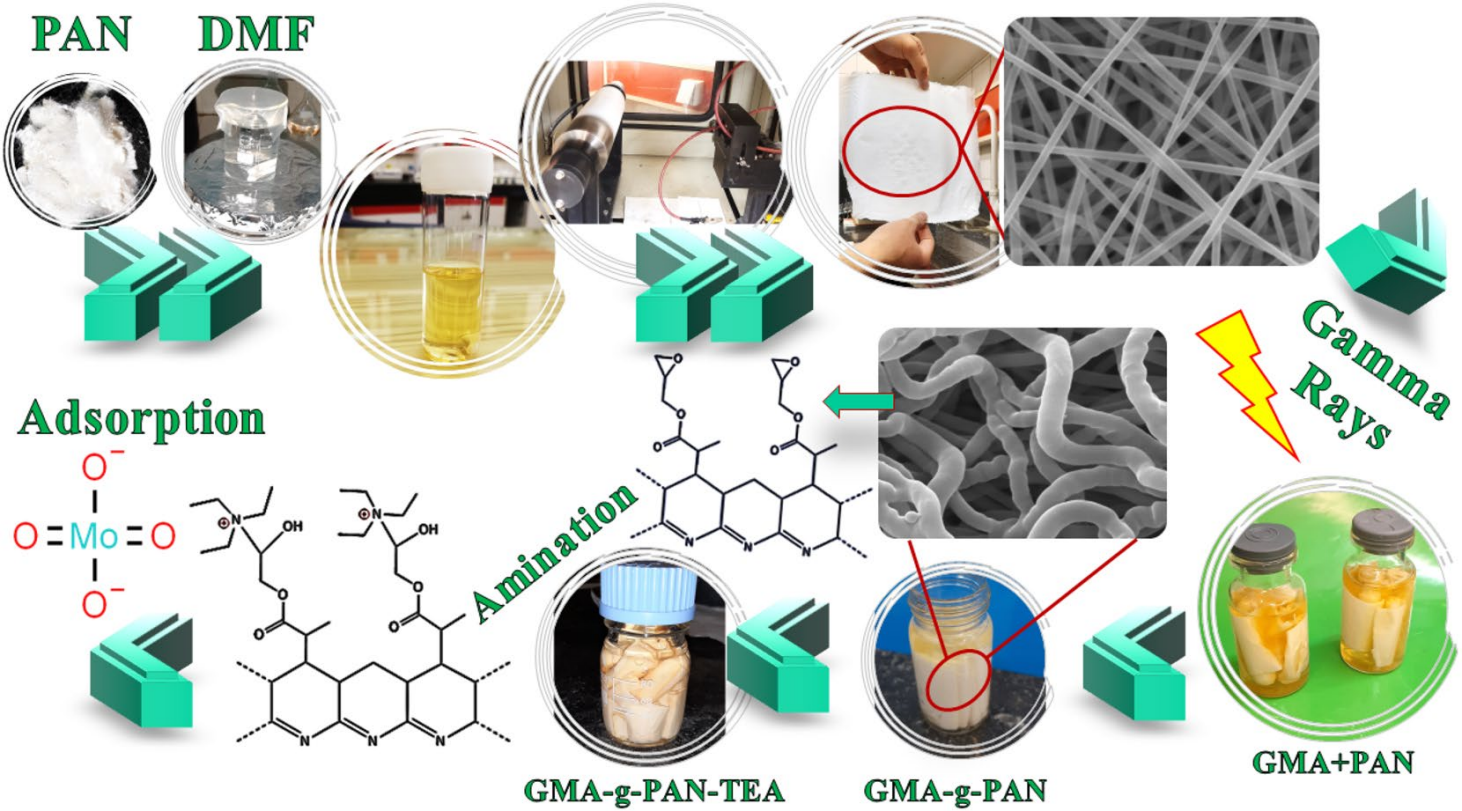
Schematic procedure for grafting and functionalization.

The samples were extracted, repeatedly washed with DI water to eliminate any remaining amines, and dried at 313 K for 24 h in the oven. The samples were then weighed. DA (degree amination) was computed with Eq. (2)^57,58^:

2 $$ DA = \frac{{\left( {\frac{{W_{a} - W_{g} }}{{M_{wa} }}} \right)}}{{\left( {\frac{{W_{g} - W_{0} }}{{M_{wm} }}} \right)}} \times 100 $$

where $W_{0}$, $W_{g}$, and $W_{a}$ represent the samples' weights before grafting, after grafting, and after amination, respectively. The amine agent's molecular weight is $M_{wa}$, while the monomer's molecular weight is $M_{wm}$ ($M_{wGMA} = 142.15 g/mol$). The schematic procedure for grafting and functionalization is shown in Fig. 1.

### Experimental design

#### RSM

The Response surface methodology (RSM) is one of the methods for getting the best function of the design parameters' maximum or lowest might be optimal. An experimental design approach must be cost-effective to extract the most detailed information possible, significantly shorten the testing time, and reduce the expense of materials and personal costs^59^. RSM is a statistical technique been frequently used for optimizing the process factors of adsorption. It is based on the multivariate non-linear model^60^, in addition, RSM entails designing experiments that will yield adequate and trustworthy measurements of the response, creating a mathematical model that fits the data obtained from the experimental design the best, and figuring out the ideal value of the independent variables that will result in a maximum or minimum response^61^. It helps research how the different process factors interact with one another. RSM analyzes the responses of several variables by concurrently altering them in a few studies. It is important to note that the response surface approach only determines the impacts of variables upon response and the interactions between the factors, not the mechanism of the processes under study. However, the RSM is an effective tool for statistical modeling and optimization of the separation processes utilizing fewer planned experimental runs than required by the experimental design^62^.

In the current study, analysis of variance (ANOVA) was used in the present research to show that the model was appropriate, and the coefficient of determination $R^{2}$ was used to show that the model was valid. Model terms are essential when the probability of the F statistic is less than 0.05, or "Prob > F" values^63^. The optimization method of the first experiment design considered the effects of monomer concentration, radiation dose, and amine concentration on the adsorption capacity and degree of grafting in the adsorption process, in addition, the optimization method of the second experiment, the effects of pH, molybdenum solution concentration, and adsorbent mass on the adsorption capacity during the molybdenum adsorption process were investigated, the explored ranges for each parameter are listed in Tables 1 and Table 2. The CCD provided 17 experimental runs for each experimental design. These runs are required to reach the optimal adsorbent performance in terms of molybdenum adsorption under the aforementioned circumstances above and to create a simulation model for this process^64^.

**Table 1:** Ranges of the studied parameters in the first experiment design.

Parameters	Unit	Ranges				
		− 2(-$\alpha )$	− 1	0	+ 1	+ 2($+ \alpha )$
GMA concentration	% (v/v)	10	15	20	25	30
Radiation dose	kGy	10	20	30	40	50
Amine concentration	%(v/v)	20	40	60	80	100

**Table 2:** Ranges of the studied parameters in the second experiment design.

Parameters	Unit	Ranges				
		− 2(-$\alpha )$	− 1	0	+ 1	+ 2($+ \alpha )$
pH	Unitless	2	3.5	5	6.5	8
Adsorbent mass	g	0.05	0.1	0.15	0.2	0.25
Mo(VI) concentration	mg/L	20	40	60	80	100

### Adsorption experiments

The flasks (50 mL) were used for the batch adsorption studies. Each flask contained 50 mg.$L^{ - 1}$ of the molybdenum solution made with ${\text{Na}}_{2} {\text{MoO}}_{4} .2{\text{H}}_{2} {\text{O}}$, and 0.1 g of the adsorbent was added. The flask was then agitated in a thermostatic shaker at 298 K and 200 rpm for four hours (well above the adsorption equilibrium time of about 1 h). Following an experiment, the adsorbents were separated, washed with DI water, and then ready for a desorption experiment.

A spectrophotometer UV/Vis (DR 6000) was used to quantify remaining Mo(VI) content in the aqueous solution. The fibers utilized in all of the adsorption experiments were from the same batch of samples, indicating that although we may disregard the level of grafting and the speed of amination, the adsorption capacity and adsorption efficiency are important criteria that should be taken into account. The adsorption capacity (q; mg.$g^{ - 1}$) and adsorption efficiency (E;%) were calculated using the following Eq.^65^:

3 $$ q = \left( {C_{0} - C_{e} } \right) \times \frac{V}{m} $$

4 $$ E\left( \% \right) = \frac{{C_{i} - C_{e} }}{{C_{i} }} \times 100 $$

where *m* (*g*) relates to the mass of the dry adsorbent and *V* (*L*) relates to the volume of Mo(VI) solution, $C_{i}$ relates to the initial concentration of molybdenum and $C_{e} \left( {mg.L^{ - 1} } \right)$ relates to the equilibrium concentrations of Mo(VI).

### Desorption experiments

Adsorbents were washed with DI water once adsorption equilibrium was established to remove any remaining solution, and they were subsequently dried in an oven for 24 h at 308 K. Utilizing a 0.5 M HCl aqueous solution, metal ions were desorption. The flasks' contents were shaken for 4 h at 200 rpm and 298 K. By using a UV spectrophotometer, the ion concentration in the solutions was evaluated. The desorption ratio (D, in%) was computed^66^:

5 $$ D\left( \% \right) = \frac{{\left( {mg\, of\, metal\, ion\, desorbed} \right)}}{{\left( {mg\, of\, metal\, ion\, adsorbed\, onto\, fiber\, mats} \right)}} \times 100 $$

### Adsorption kinetic

An amount of adsorbent (Amine-GMA-g-PAN) was used in a 50 mL molybdenum solution with an initial concentration of 30 mg/L for the adsorption kinetic measurements. At temperatures ranging from 298 to 323 K, the volumetric flasks were stirred for periods ranging from 0 to 180 min. Mo(VI) concentration was determined throughout a range of time intervals. Pseudo-first-order, pseudo-second-order, and Weber–Morris kinetic models were used to examine the data^67^.

### Adsorption isotherm

The adsorption isotherms were investigated by agitating the amount of adsorbent with 50 mL of molybdenum solution (20, 40, 60, 80, and 100 mg/L) for 24 h at various temperatures (298, 323, and 338 K). To examine the isotherm data, the Langmuir, Freundlich, and Dubinin-Radushkviech (D-R) models were employed.

### Thermodynamic

At different temps of 298, 308, 318, and 328 K, the adsorption of a molybdenum solution was examined in a mixer for 240 min at a speed of 200 rpm. $\Delta H^{0}$, $\Delta S^{0}$, and $\Delta G^{0}$ were used to identify the nature and spontaneity of the adsorption process using thermodynamic parameters.

### Selective adsorption

The capacity of the adsorption system to extract a metal ion (like molybdenum) from a mixture of metal ions is measured by the separation coefficient. The term "selectivity" is sometimes used to refer to the separation factor. It may also be stated as a ratio of the distribution coefficients of the ions to be separated and is used to measure the probability of chromatographic separation.

If Mo(VI) is chosen as the preferable adsorbent, then the value of $\alpha_{Al, Fe,Cu, \ldots }^{Mo}$ is greater than 1. In contrast, $\alpha_{Al,Fe,Cu, \ldots }^{Mo}$ < 1 indicates that other metal ions are favored.

6 $$ separation\,factor \left( {\alpha_{Al,Fe,Cu, \ldots }^{Mo} } \right) = \frac{{K_{d} \left( {Mo} \right)}}{{K_{d} \left( {Al,Fe,Cu, \ldots } \right)}} $$

7 $$ K_{d} \left( {mL/g} \right) = \frac{{C_{o} - C_{e} }}{{C_{e} }} \times \frac{V}{m} $$

where $K_{d} \left( {Al,Fe,Cu, \ldots } \right)$ and $K_{d} \left( {Mo} \right)$ are the distribution coefficients of the several competing Mo(VI) and other metal ions species in the adsorption system and *m* (*g*) relates to the adsorbent mass and *V* (*L*) denotes the volume of the solution in the adsorption test^68^.

## Instruments and techniques

### UV spectrophotometer

The absorbance at wavelengths of 630 or 635 nm was used to calculate the concentration of the Mo(VI) solution. At room temperature (298 K), optical density measurements were performed using a single-beam UV–VIS (DR6000) spectrophotometer and a quartz cell with a 1 cm optical length.

#### ICP

The concentration of Mo(VI) ions with other ions were measured using inductively coupled plasma mass spectrometry.

#### SEM

ZEISS EVO18 Scanning Microscope (special edition, Germany) was used for scanning electron microscopy measurements.

#### ATR-FTIR spectroscopy

Analysis by infrared spectroscopy was carried out using a vertex 70, Bruker spectrometer, in the range from 500 to 4000 ${\text{cm}}^{ - 1}$.

#### Brunauer, emmett and teller (BET)

To calculate the pore volume, surface area, and pore size distribution of the adsorbent, BET is used with $N_{2}$ adsorption–desorption at cryogenic temperatures and under vacuum. The particular surface area and porous characteristic are significant factors affecting how well nanofibers adsorb substances.

## Results and discussion

The PAN nanofiber samples were exposed to $\gamma$-radiation in the GMA monomer solution using a simultaneous irradiation technique. On the polymeric chain, this causes radical formation, and the locations of the radical formation serve as the starting places for the side chains. A combination of graft copolymer and homopolymer was produced due to radiation-induced polymerization of the monomer, which co-occurred, coincided^69^. To decrease the production of homopolymer reactions in the monomer solution, ferrous sulfate heptahydrate salt (0.5% wt%) was utilized as inhibitor salt. The homopolymer and the unreacted monomer were then thoroughly rinsed off the grafted PAN nanofiber with methanol. The created polymer itself causes radiolysis, and the radicals produced on nearby polymer chains may react with one another to form a covalent connection, linking the polymer molecules and creating a three-dimensional network structure that results in a crosslinked polymer. The concentration of monomer, type of solvents, and radiation dose are the main factors that affect the grafting yield^70^.

**Figure 2:**
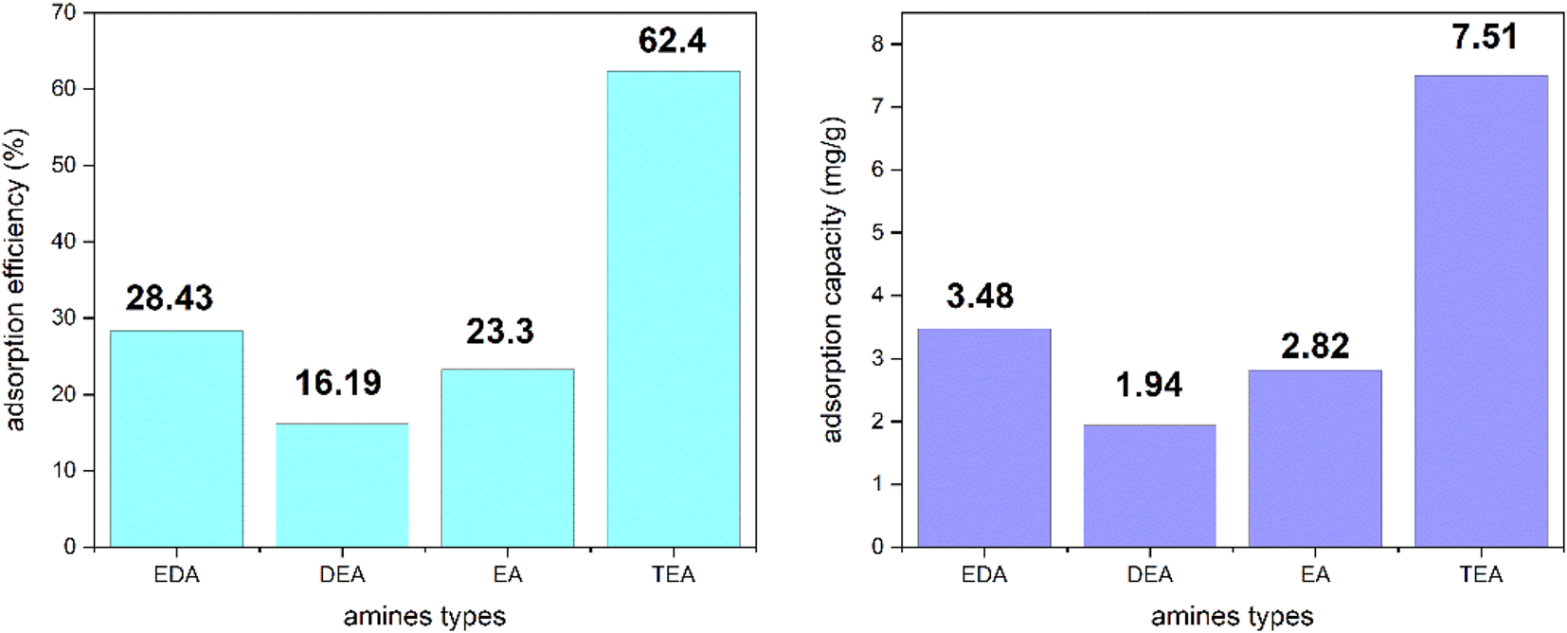
Preliminary investigation of different amines in molybdenum adsorption.

Electrospun PAN nanofibers coated with GMA monomers were individually modified with a variety of amines, including diethylamine, ethylenediamine, triethylamine, and ethanolamine, in the early experiments for determining the type of amine. In order to choose the final change based on the greatest adsorption, each adsorbent was then evaluated for molybdenum adsorption under the exact same circumstances, such as 15%(v/v) GMA, 20 kGy irradiation dosage, and 60%(v/v) amine content. Figure 2 displays the adsorption capacity (mg/g) and adsorption efficiency (%) for adsorbents using various amines.

As can be seen, adsorption capacity(mg/g) and adsorption efficiency (%) for modification with triethylamine are higher than other amines, because only the reaction of the tertiary amine with GMA causes a positive charge on the adsorbent surface and it can adsorb molybdate.

### Effect of different factors on the degree of grafting and adsorption capacity

The objective is to determine and examine the factors influencing the outputs using the fewest possible experiments. The response surface methodology (RSM) is one of the strategies for designing experiments. They cannot accurately represent many processes analytically because of the large number of control variables and computing complexity. In these situations, using experimental modeling techniques is helpful.

RSM is a mathematical and statistical approach to process optimization. RSM typically includes three steps: design and experimentation, regression modeling of the response surface, and optimization come first. RSM's primary goal is to choose the best state or range to satisfy the test requirements. The independent parameters and the response are associated with the response level technique as follows:

8 $$ y = f\left( {x_{1} ,x_{2} ,x_{3} , \ldots .x_{n} } \right) \pm e $$

In the equation above, *f* stands for the answer function, *y* for the answer, *e* for th*e* test error and $x_{1} , x_{2} , x_{3}$, etc. are also independent variables.

We get a level known as the response level when we draw the replies. Depending on how the curve is shaped, the figure can be a first-order polynomial or a higher-order polynomial. Adsorbent synthesis conditions were initially optimized, with monomer content, radiation dosage, and amine concentration being treated as separate input variables to improve grafting and adsorption capacity. Total of 17 experiments was conducted in the current investigation using three independent variables. To research the concurrent influence using the CCD in the RSM, the variables of monomer concentration, irradiation dosage, and amine concentration in molybdenum adsorption from the aqueous solution were examined (as shown in Table A in supplementary file).

A link between independent input variables and responses is empirically shown when the response level model, the following are these equations:

9 $$ \left( {degree\, of\, grafting } \right) Y_{1} = + 354.65 + 2.21A - 13.47B + 2.69C + 0.37AB - 0.043AC + 0.0071BC + 0.375A^{2} + 0.1105B^{2} - 0.0082C^{2} $$

10 $$ \left( {adsorption\, capacity} \right) Y_{2} = - 18.21 + 1.02A + 0.796B + 0.669C - 0.023AB - 0.0037AC - 0.00199BC - 0.0077A^{2} - 0.0029B^{2} - 0.0052C^{2} $$

The independent input variables are A (monomer concentration), B (irradiation dosage), and C (amine concentration). Table B (supplementary file) shows the analysis of variance (ANOVA) findings for the quadratic model for adsorption capacity. The $R^{2}$ and its modified $R^{2}$ values are very near to one (see Table C in supplementary file). This number represents the similarity of the observed and expected values. It implies that the regression model shows how the specified independent variables and the provided responses relate to one another. The model's Prob. > *F* value is < 0.0001, which shows that it is statistically valid. The lack-of-fit term is not significant (more than 0.05) as it is desired. The quadratic model was shown to be appropriate for the current investigation by the substantial value of lack of fit (greater than 0.05). The quadratic model is statistically suitable for the replies.

**Figure 3:**
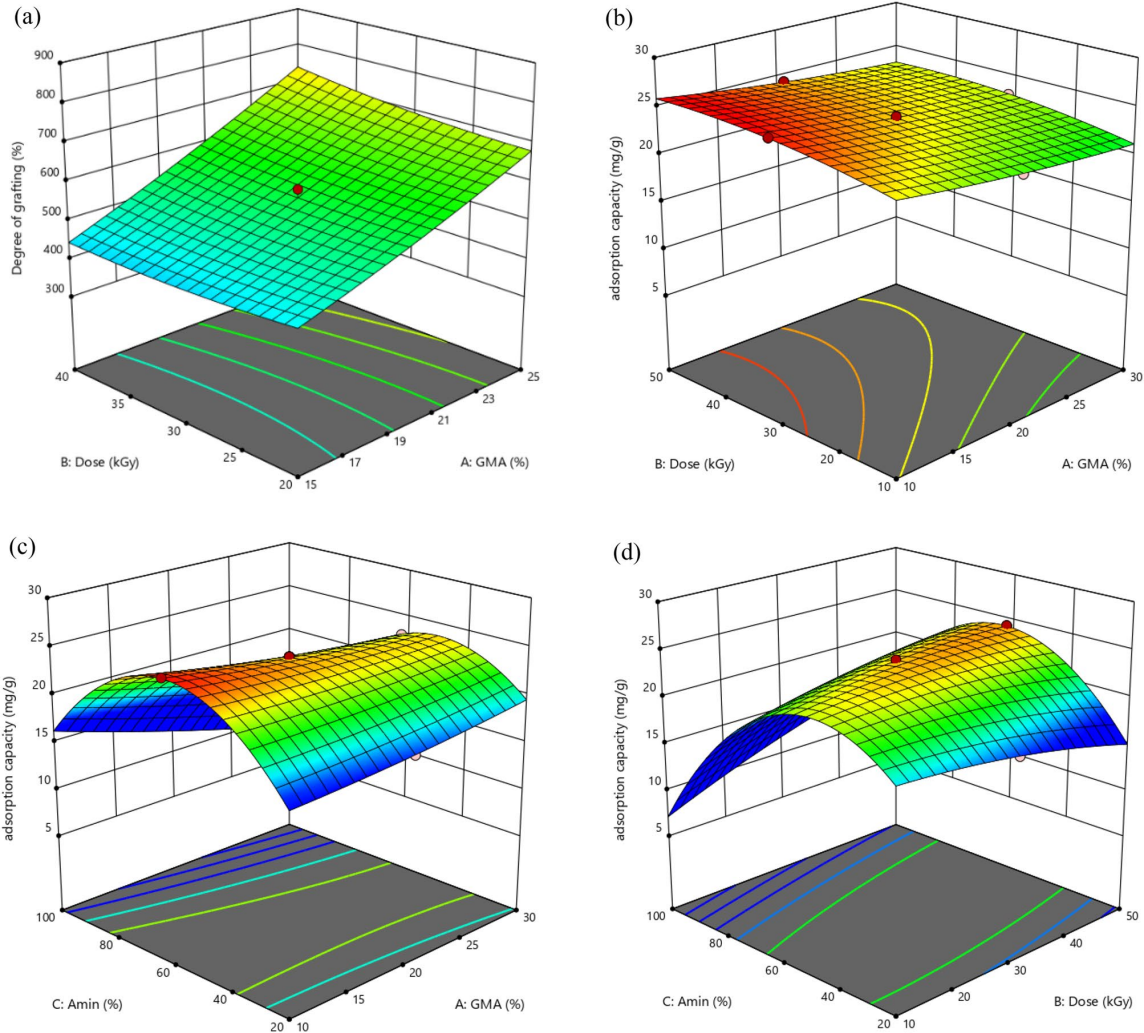
3D plot (**a**) showing effect of GMA concentration and irradiation dose on degree of grafting, (**b**) showing effect of GMA concentration and irradiation dose on adsorption capacity, (**c**) showing effect of GMA concentration and amine concentration on adsorption capacity, (**d**) showing effect of amine concentration and irradiation dose on adsorption capacity, (Mo (VI) ions aqueous solution = 50 mg/L).

Figure 3 illustrates how the outcomes are shown as 3D-lines and graphs. The defined range for the concentration of GMA is 10–30%, the defined range for the irradiation dose is 10–50 kGy, and the defined range for the concentration of amine is 20–100%. However, the change in grafting degree at 326–858% was influenced by the factors of monomer concentration and irradiation dose. The degree of grafting could be dramatically improved by increasing the monomer concentration and irradiation dose, but an excessively high monomer concentration had the opposite effect on adsorption capacity because more homopolymer formed in the reaction solution and could cover the active sites on the adsorbent. In a particular range of amine concentration, as amine concentration rises, the amount of adsorption increases to a certain extent and then decreases because the amount of amine in the solution affects both the chemistry of the solution and the activity of the functional groups on the adsorbent surface^71,72^. So, for the adsorption of molybdenum from a solution, there is a range of monomer content, irradiation dosage, and amine concentration that is advantageous. Optimized process variable values for the adsorption process in the first experiment design is shown in Table 3.

**Table 3:** Optimized process variable values for adsorption process in the first experiment design.

GMA concentration %	Irradiation dose (KGy)	Amine concentration %	Degree of grafting	Adsorption capacity mg/g
10%	34.38	60.97%	331.97	22.09

According to the findings, it is shown that monomers are swiftly bound on the surface of the polymer adsorbent when utilizing the simultaneous irradiation approach. This very effective adsorbent may be readily extracted from the solution and suggested as a potential use in the industry^73^.

The parameters of the adsorption conditions, such as the pH of the solution, the initial concentration of the molybdenum, and the mass of the adsorbent, were optimized in another experiment after the parameters of the adsorbent synthesis conditions were determined to have the best values. The solubility of the metal ion and the overall charge on the adsorbent surface are both impacted by the pH value and impact the adsorption capacity. The experimental data from 17 runs of RSM is shown in Table D (supplementary file).

**Figure 4:**
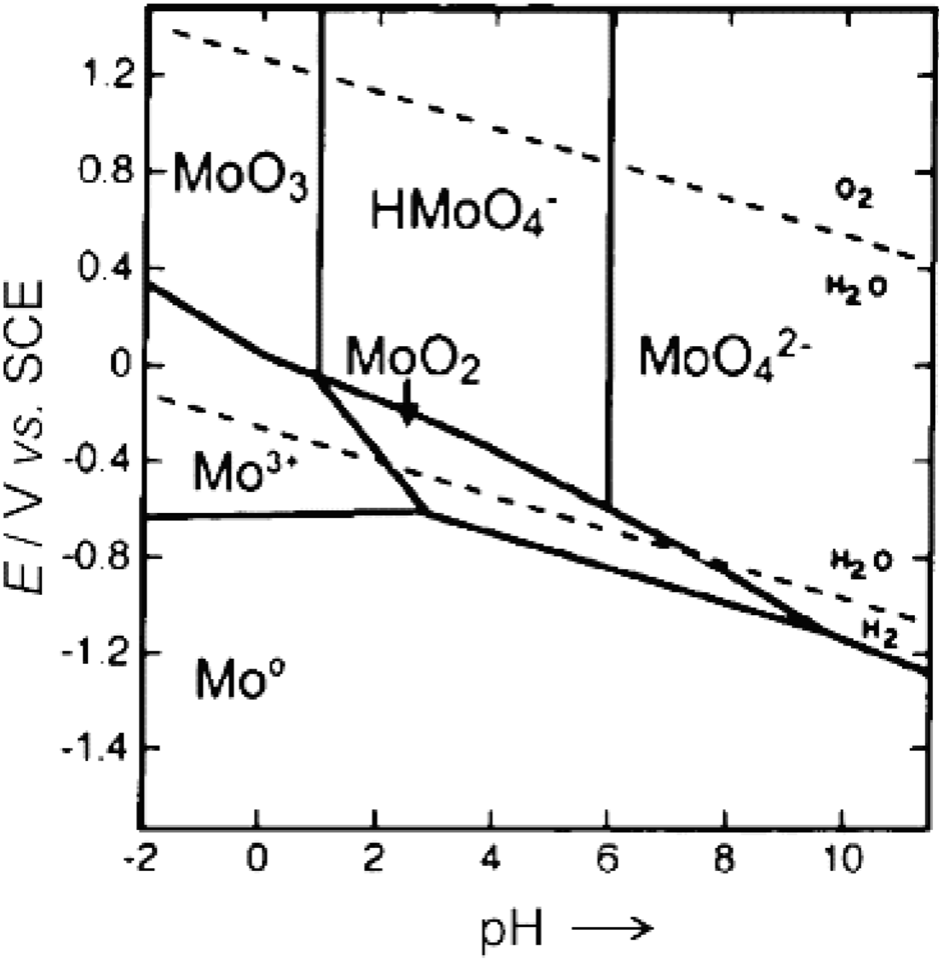
Pourbaix diagrams for molybdenum.

Pourbaix diagrams showed the isothermal phase equilibrium of a specific element in contact with water in redox potential-pH space^74^. According to the Pourbaix diagram for molybdenum (Fig. 4), at pH > 2 and zero potential, molybdenum is in the forms of ${\text{HMoO}}_{4}^{ - }$ and ${\text{MoO}}_{4}^{2 - }$ and is soluble in water. On the other hand, the point of zero charge (${\text{pH}}_{PZC}$) value of adsorbents can be used to discuss the impact of solution pH on the adsorption process.

**Figure 5:**
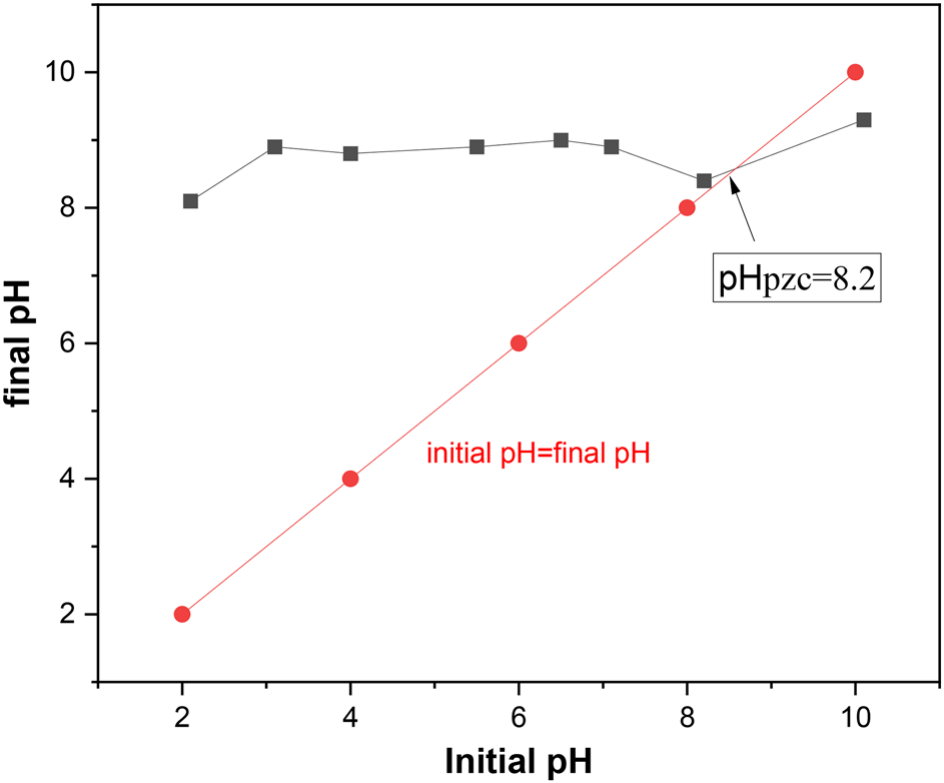
$$pH_{pzc}$$ of adsorbent.

The point of zero charge (${\text{pH}}_{PZC}$) explains the situation where there is no electrical charge accumulation on the surface (zero charge) and explains the adsorption process related to the adsorbate surface charge. At lower pH (acidic medium), the surface of the nanofiber (below ${\text{pH}}_{{{\text{PZC}}}}$) is positively charged and attracts negatively-charged molecules, which increases the adsorption of molybdenum and at higher pH (above ${\text{pH}}_{PZC}$) the surface of the nanofiber is negatively charged and attracts positively charged molecules in solution, which decreases the adsorption of molybdenum^67,75^ and the ${\text{pH}}_{PZC}$ of the adsorbent was measured using the pH drift method and was found to be 8.2 as shown in Fig. 5. These findings demonstrate that cationic functional groups are present on the surface of the adsorbent. The chosen range for pH is between 2 and 8 because a positive charge of the adsorbent may be produced in pH environments below $H_{PZC}$, preferring the adsorption of negatively charged species such as Mo anionic species.

**Figure 6:**
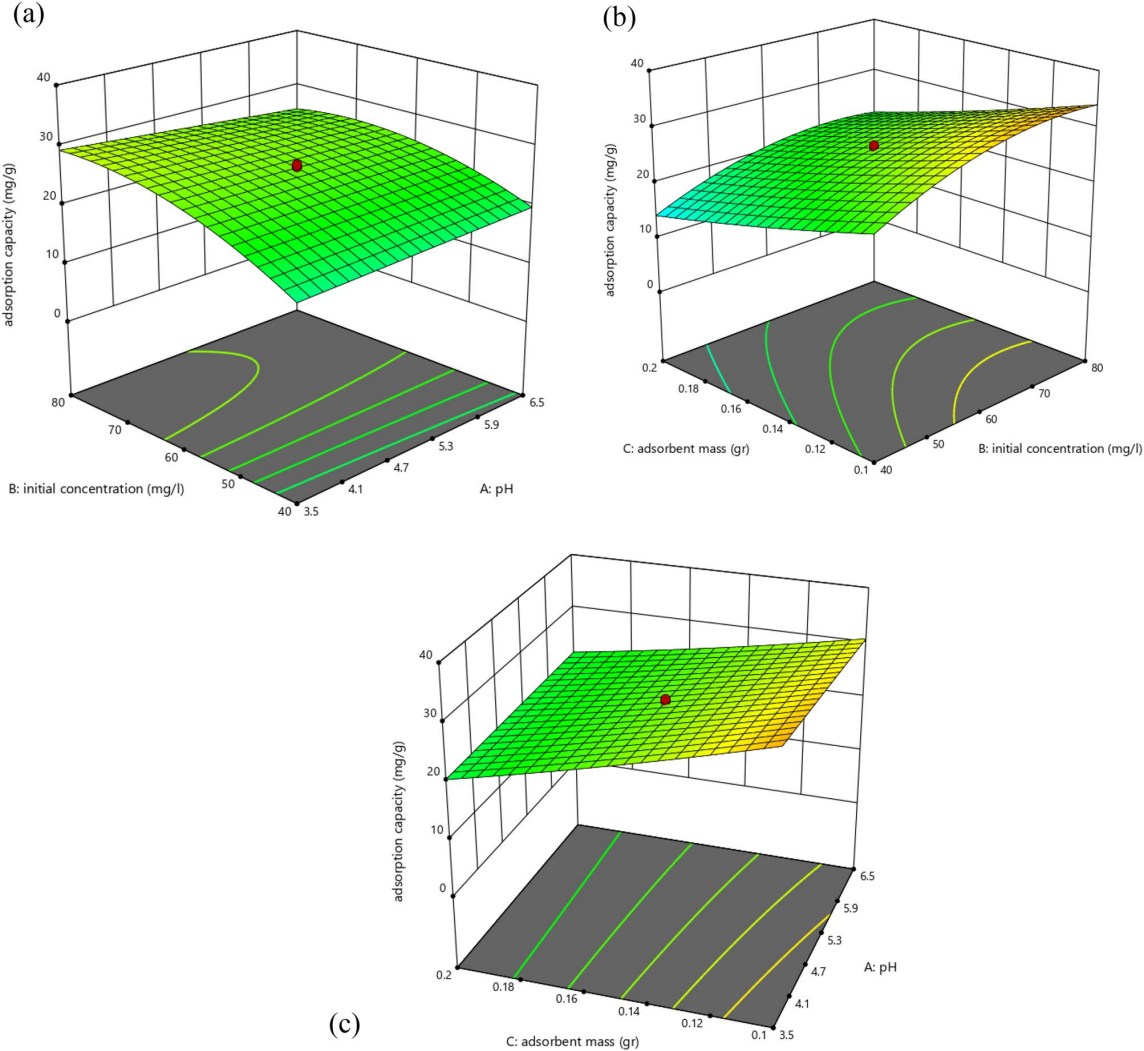
3D plot (**a**) showing effect of initial concentration and pH on adsorption capacity, (**b**) showing effect of initial concentration and adsorbent mass on adsorption capacity, (**c**) showing effect of pH and adsorbent mass on adsorption capacity.

Figure 6 illustrates the outcomes of operating parameters as 3D graphs. The initial concentration of molybdenum in design expert was considered in the range of 20–100 mg/L and increasing or decreasing the initial concentration of the metal ion can have an effect on the adsorption process using polymer nanofibers. Increasing the initial concentration can initially enhance the adsorption capacity by the polymer nanofibers, however, at very high concentrations, the absorption capacity of the polymer may get saturated and may not efficiently adsorb the ions. On the other hand, decreasing the initial concentration of the metal ion may lead to a lowered overall adsorption capacity, or it may not be cost-effective. In other words, the initial concentration of the metal ion needs to be carefully chosen to achieve optimal adsorption performance using polymer nanofibers^76^.

In terms of mass transfer, larger concentrations improve the rate of adsorption because, at higher starting concentrations, the high concentration gradient between the soluble mass and the adsorbent's external surface enhances the transfer of foreign mass^77^.

The amount of adsorbents supplies the Mo adsorption active sites and establishes the molybdenum adsorption potential at a certain starting concentration. in constant initial concentration, less adsorbent leads to higher adsorption capacity and, typically, poorer adsorption efficiency. The area of the adsorbent surface grows with an increase in adsorbent quantity, increasing the amounts of active sites that are accessible, so the range of 0.05–0.25 g was determined for the adsorbent mass.

Table E (supplementary file) provides the analysis of variance (ANOVA) finding for the quadratic model for adsorption capacity. When using the RSM, which is based on parameter prediction, a relationship between independent input factors and responses is experimentally shown. The following are these equations:

11 $$ \left( {adsorption\, capacity} \right)Y_{1} = 3.59422 + 0.013110A + 1.40859B - 215.71567C - 0.042069AB + 14.49078AC - 0.392916BC - 0.005230A^{2} - 0.007748B^{2} + 174.17613C^{2} $$

The independent input variables, pH, initial concentration, and adsorbent mass, have coefficients A, B, and C, respectively. According to simulation software, Table 4 shows the perfect settings for the adsorption. The acquired experimental findings validate the ideal software adsorption conditions. This table demonstrates the substantial adsorption property and excellent surface optimization of the produced adsorbent.

**Table 4:** Optimized process variable values for adsorption Mo(VI).

	GMA con. %	Irradiation dose (kGy)	Amine con.%	pH	Initial con. Mo (mg/L)	Adsorbent mass (g)	Degree of grafting%	Adsorption capacity mg/g
Software	10	34.38	60.97%	3.5	70	0.06	331.97	53.02
Experimental	10	30	60	3.2	68.75	0.0652	326.83	50.75

### Characterization

FTIR, SEM, and BET experiments were used to characterize and validate the produced polymer adsorbent.

#### FTIR analysis

**Figure 7:**
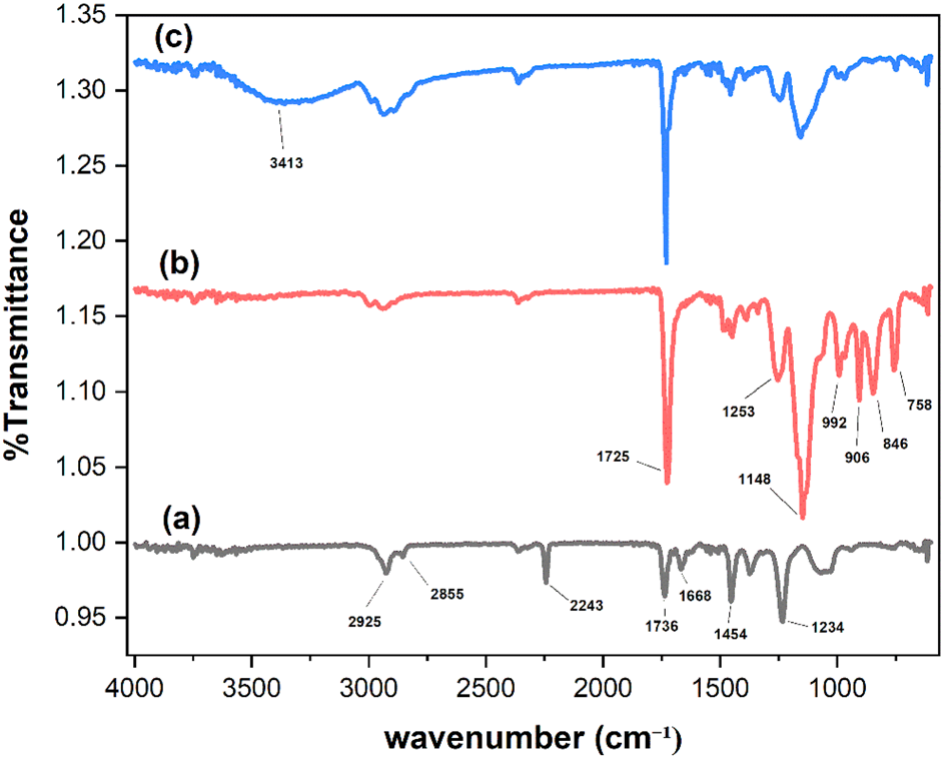
FTIR analysis: (**a**) electrospun PAN, (**b**) GMA grafted onto the PAN fiber, (**c**) amination GMA-g-PAN.

By using the FT-IR (ATR method), the chemical composition of nanofiber membranes was examined. Figure 7a illustrates the distinctive peak of PAN reflecting the stretching vibration of nitrile groups ($$- C \equiv N$$), which was detected at 2243 $${\text{cm}}^{ - 1}$$, and the peaks at 2925 and 2855 $${\text{cm}}^{ - 1}$$ attributable to the $$- {\text{CH}}$$, $$- CH_{2}$$ and $$- {\text{CH}}_{3}$$ stretching vibrations. At 1454 $${\text{cm}}^{ - 1}$$ and 1668 $${\text{cm}}^{ - 1}$$, the $$- {\text{CH}}$$ bending vibrations and amide group associated with DMF were detected^78^. Additionally, the stretching vibrations of the C = O and C–C created the characteristic peaks at 1736 and 1234 $${\text{cm}}^{ - 1}$$, respectively. Figure 7b shows the FTIR spectrum of the GMA grafted onto the PAN nanofiber (GMA-g-PAN) changed obviously; at 2243 $${\text{cm}}^{ - 1}$$ disappeared from the characteristic peak of the nitrile group ($$- C \equiv N$$) and reaction with GMA, new adsorption peaks of 1725, 1148 and 992 $${\text{cm}}^{ - 1}$$ are corresponding to the stretching vibrations of the carbonyl group of the ester of the GMA, C–O–C and double bond of GMA, respectively^79^. In addition, the epoxy groups of GMA are responsible for the appearance of additional adsorption peaks at 1253, 906, 846, and 758 $${\text{cm}}^{ - 1}$$. These peaks could be seen in GMA-g-PAN, proving that GMA was successfully grafted onto the PAN fiber. After amination as shown in Fig. 7c, the typical peaks of the GMA epoxy group disappeared, in the modification process, TEA molecules attach to the epoxy groups on the grafted GMA chains of the fibers, forming a covalent bond. This results in the opening of the epoxy ring in different solvents, as the epoxy ring is highly conducive to the covalent immobilization of amine molecules^80^ and converting the epoxy group to an alcohol group in the polymeric chains and appeared the peak at 3413 $${\text{cm}}^{ - 1}$$ ($$O - H)$$^81^.

#### SEM images

**Figure 8:**
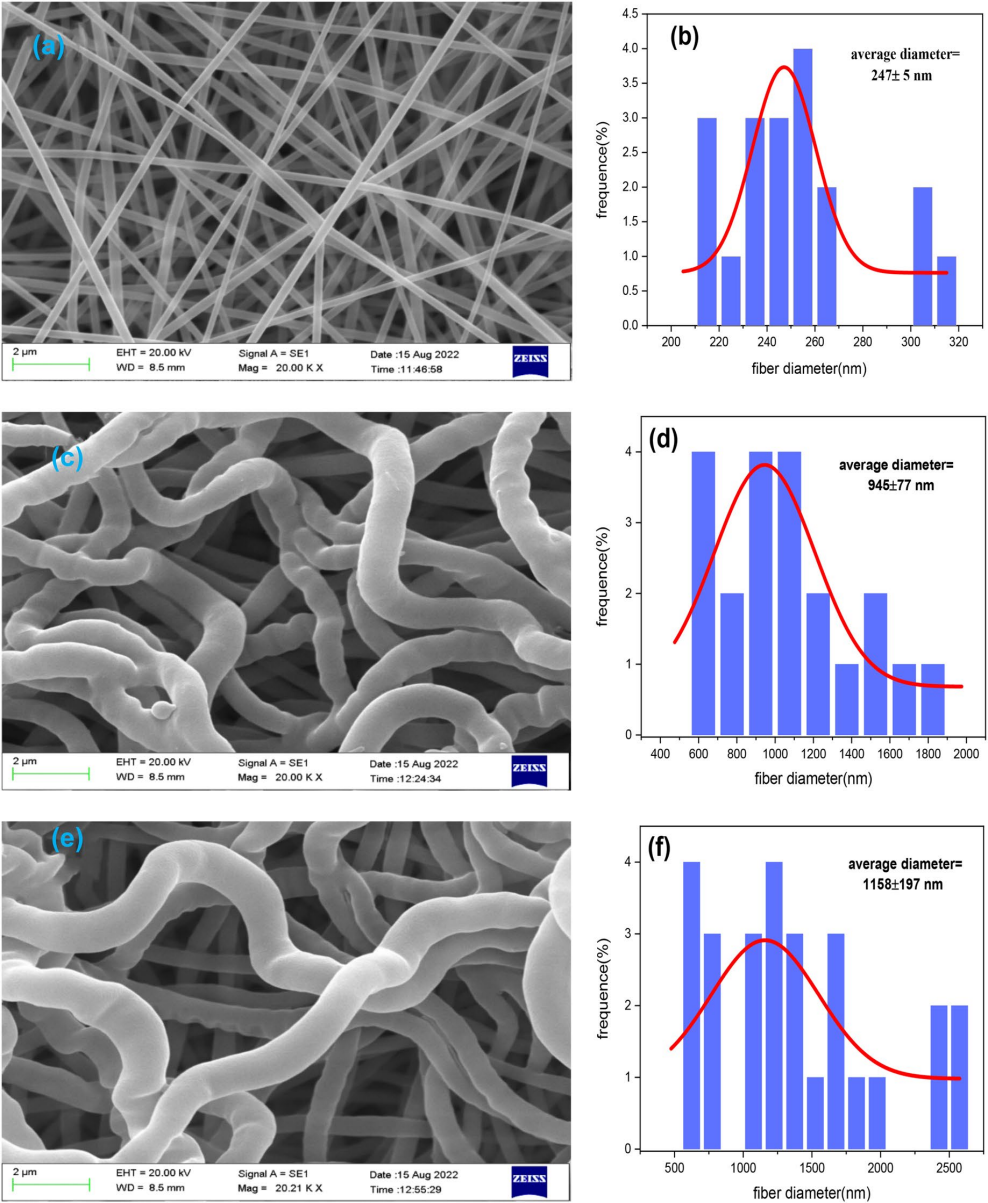
SEM images and the diameter distributions of the nanofiber. (**a**) and (**b**) PAN, (**c**) and (**d**) GMA-g-PAN, (**e**) and (**f**) Amine-GMA-g-PAN.

Many researchers use SEM to examine the morphology of the nanofibers in polymer research and better to understand the structural features of polymers. The morphology acquired by SEM is shown in Fig. 8. The PAN solution (15 wt%) was electrospun to provide the homogeneous and smooth surface of the nanofiber (Fig. 8a) and the average diameter of pure PAN was 247 ± 5 nm (Fig. 8b). After the polymerization procedure, PAN nanofibers were grafted with GMA, increasing the nanofibers' diameter (945 ± 77 nm), as illustrated in Fig. 8d. The fiber became thick and coarse due to a heterogeneous grafting layer forming on its surface^82^. The surface of GMA-g-PAN was shown in Fig. 8c to be rougher than pure PAN. A rough surface may enhance the nanofiber's capacity to cling to it. The aminated GMA-g-PAN nanofiber mats formed after the modification procedure with triethylamine for adsorption Mo(VI) are 1158 ± 197 nm in diameter, resulting in the average diameter greater than that of PAN and GMA-g-PAN as shown in Fig. 8e f. Further Additional physical evidence for grafting and modification includes changes in physical appearance and an increase in average fiber diameter after GMA and amine modification.

#### BET analysis

**Figure 9:**
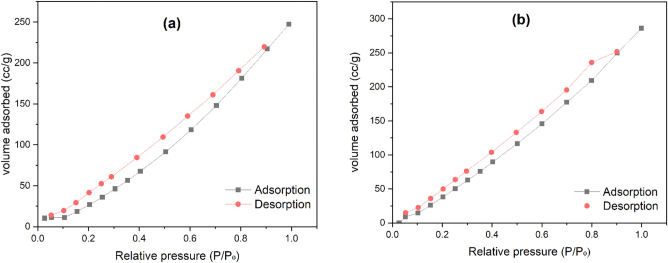
$N_{2}$ adsorption–desorption isotherms for (**a**) electrospun PAN and (**b**) modified PAN (Amine-GMA-g-PAN).

The shape of the adsorption isotherm may be used to get qualitative information about the adsorption process and surface area. According to the IUPAC category, there are six fundamental kinds of adsorption isotherms (Types I–VI). Figure 9a,b, which represent electrospun PAN of types V and modified PAN (TEA-GMA-g-PAN) of types IV, respectively^83^. Mesoporous materials often exhibit Type IV isotherms. The hysteresis loop, which is connected to the occurrence of pore condensation, is the most distinctive aspect of the type IV isotherm. A plateau of the isotherm occurs from the limiting uptake across a range of high $P/P_{0}$, which denotes complete pore filling. Similar to the type II isotherm, the first part of type IV may be attributable to monolayer-multilayer adsorption. Pore condensation and hysteresis may be seen in type V isotherms. The first part of this adsorption isotherm, in contrast to type IV, is connected to type III adsorption isotherms, showing relatively modest attractive interactions between the adsorbent and the adsorbate. The pore diameters are categorized by the IUPAC into three general categories: micropores (*d* < 2 nm), mesopores (2 < *d* < 50 nm), and macropores (*d* > 50 nm)^84^. The mesoporosity on the surface of the adsorbent is indicated by the hysteresis. Throughout the entire relative pressure range, the quantity of nitrogen absorbed on the adsorbent steadily rises, suggesting that a limited number of mesopores are present in this adsorbent. The hysteresis loop of the mesopores isotherm, which is connected to capillary condensation occurring in mesopores, is one of its distinguishing characteristics. The pore size distribution seems to be located in the mesopore region^85^. The pore characteristic of electrospun PAN and modified PAN (adsorbent) is summarized in Table 5, the adsorbent surface area is 529.472 $m^{2} /g$, adsorbent has 0.592 cc/g of pore volume and 2.198 nm of pore diameter calculated by the BJH method for the synthesized samples.

**Table 5:** $N_{2}$ adsorption/desorption analysis of electrospun PAN and adsorbent.

Parameter	Electrospun PAN	Amine-GMA-g-PAN
Surface area	382.933 m^2^ /g	529.472 m^2^ /g
Pore volume	0.493 cc/g	0.592 cc/g
Pore diameter	2.476 nm	2.198 nm

### Adsorption

#### Adsorption isotherm model

**Figure 10:**
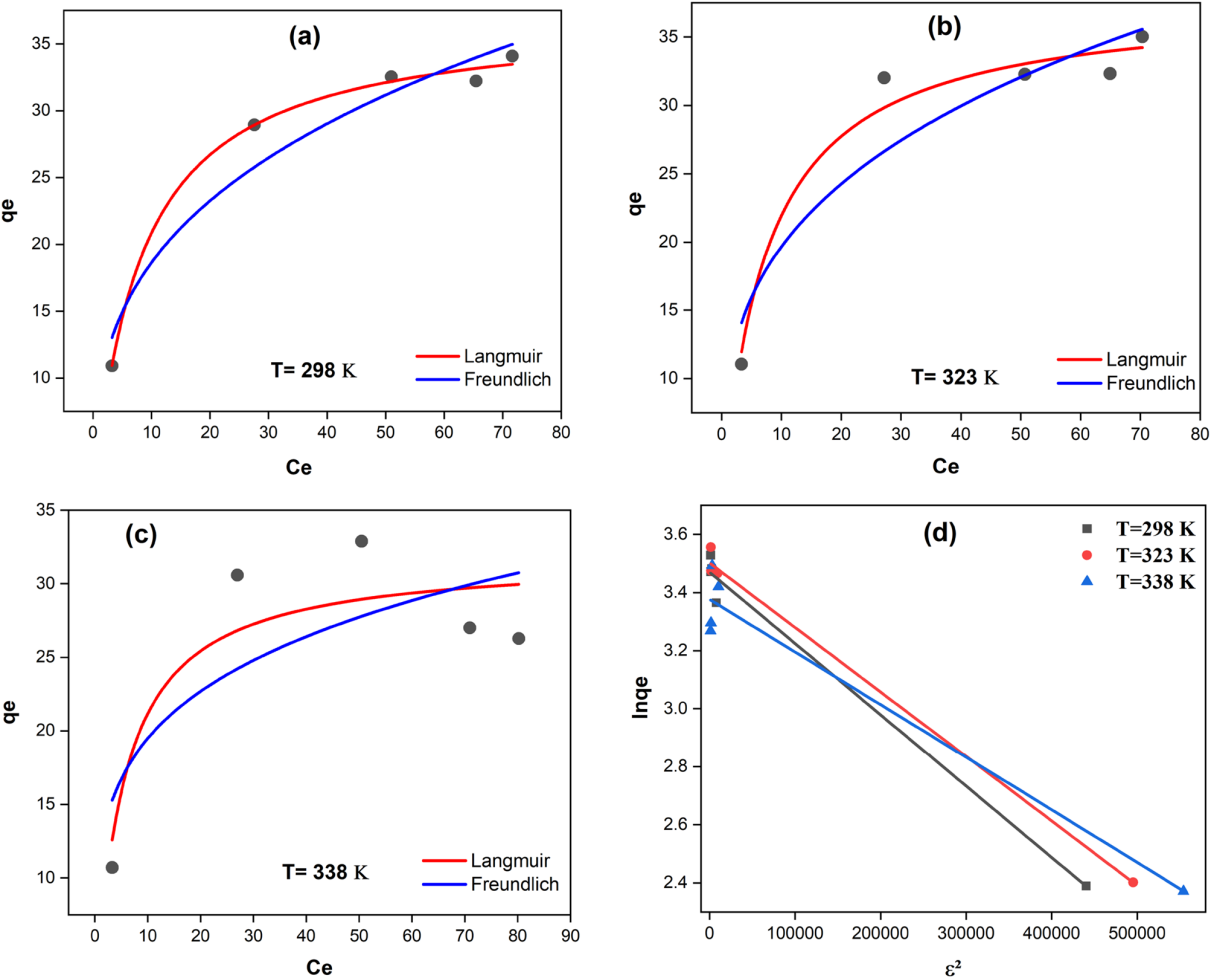
Isotherm plots for the adsorption of Mo(VI). (**a**) Langmuir, (**b**) Freundlich, (**c**) D–R.

An adsorption isotherm describes the relationship between the molybdenum concentration in the liquid and the amount that absorbed onto the absorbent at steady temperature. To achieve equilibrium between the adsorbent and residual ion concentration in the liquid phase, an exact relationship is required. Figure 10 displays the findings of our investigation into the equilibrium adsorption isotherm at three different temperatures (298 K, 323 K, and 338 K) and The initial Mo(VI) concentration was varied in the adsorption tests (20, 40, 60, 80, and 100 mg/L).

In the present research, the maximum adsorption capacity and adsorption equilibrium type were shown using the Langmuir model (Eq. 12), Freundlich model (Eq. 13) and D-R model (Eq. 14). The D-R isotherm is often used to determine the porosity and the adsorption-free energy. On the absorbent surface, the Langmuir model was linked to uniform and monolayer adsorption, while the Freundlich model was connected to multilayer adsorption. Because it does not require a homogeneous surface or a constant adsorption potential, this model is more inclusive. The following is one way to express the isotherms^86^:

12 $$ \frac{{C_{e} }}{{q_{e} }} = \frac{1}{{q_{m} K_{L} }} + \frac{{C_{e} }}{{q_{m} }} $$

13 $$ \ln q_{e} = \ln K_{F} + \frac{1}{n}\ln C_{e} $$

14 $$ ln\,q_{e} = ln\,q_{m} - \beta \varepsilon^{2} $$

where $q_{e}$ ($mg.g^{ - 1}$) is the adsorption capacity at equilibrium, $q_{m}$ ($mg.g^{ - 1}$) is the maximum adsorption capacity (theoretical isotherm saturation capacity), and $C_{e}$ is the concentration of molybdenum after adsorption ($mg.L^{ - 1}$). $K_{L}$ ($L.mg^{ - 1}$) is the Langmuir constant, $K_{F} \left( {\left( {mg.g^{ - 1} } \right)\left( {L.mg^{ - 1} } \right)^{\frac{1}{n}} } \right)$ is a Freundlich constant, n is related to the adsorption intensity. where $\beta$ is the D-R isotherm constant ($mol^{2} .kJ^{ - 2}$) and $\varepsilon$ is called the "Polanyi potential" which is described by Eq. 15:

15 $$ \varepsilon = RTln\left( {1 + \frac{1}{{C_{e} }}} \right) $$

where *R* is the universal gas constant ($8.314 J.mol^{ - 1} .K^{ - 1}$) and *T* (*K*) is the temperature. The mean free energy of sorption ($kJ.mol^{ - 1}$) is calculated using the following Eq. 16:

16 $$ E_{DR} = \frac{1}{{\sqrt {2\beta } }} $$

The mean free energy of sorption ($$E_{DR}$$), which may be physical or chemical, provides details on the sorption process. The chemical ion exchange process controls sorption when $$E_{DR}$$ is between 8 and 16 kJ/mol. If $$E_{DR} $$ is below 8.0 kJ/mol, physical forces could impact on the sorption process; if $$E_{DR}$$ is over 16 kJ/mol, particle diffusion might have an effect^87^.

According to Fig. 10 and the data of the results expressed at 298 K in Table 6, the correlation coefficient of the Langmuir isotherm model was 0.996. It showed that the adsorption progress is consistent with the Langmuir model and the adsorption process is physical and monolayer. But with the increase in temperature, the correlation coefficient of the D-R model has improved and the correlation coefficient at 323 K was 0.995. The reason may be that the rise in temperature causes the swelling of the absorbent and the increase in the volume of the porosity, after which the adsorption is controlled by molecular diffusion.

**Table 6:** Isotherm parameters for adsorption of Mo (VI) at different temperatures.

Model	Isothermal model parameters	$298 K$	$323 K$	$338 K$
Langmuir	$q_{m} \left( {mg.g^{ - 1} } \right)$	37.74	37.13	31.84
	$K_{L} \left( {L.mg^{ - 1} } \right)$	0.128	0.146	2.68
	$R_{L}$	0.348 − 0.068	0.318 − 0.06	0.025 − 0.003
	$R^{2}$	0.996	0.975	0.820
Freundlich	$K_{F} \left( {mg.g^{ - 1} } \right)\left( {L.mg^{ - 1} } \right)^{\frac{1}{n}}$	7.36	7.51	8.34
	1/n	0.3707	0.3732	0.3075
	$R^{2}$	0.946	0.886	0.615
D-R	$q_{m} \left( {mmol.g^{ - 1} } \right)$	32.14	33.12	29.25
	$\beta \left( {mol^{2} .KJ^{ - 2} } \right)$	2.45	2.22	1.81
	$E_{DR} \left( {KJ.mol^{ - 1} } \right)$	0.45	0.47	0.53
	$R^{2}$	0.9877	0.9953	0.9565

Additionally, it is discovered that the maximum adsorption capacity predicted by the Langmuir curve is 37.74 $mg.g^{ - 1}$.

As a result, it is possible to conclude that the adsorption of Mo(VI) onto an adsorbent follows the Langmuir isotherm (Table 6 includes the correlation coefficients $R^{2}$ along with the Langmuir, Freundlich, and D-R isotherm constants identified by fitting the adsorption data to these isotherms at three temperatures).

The findings also demonstrate that when the temperature falls, adsorption capacity rises. This could be because the interaction between the Mo(VI) ions and the adsorbent's active group would be more favorable at a lower temperature. As a result, raising the operating temperature over 298 K reduces the strength of the adsorbate-adsorbent interaction.

The separation factor, also known as the equilibrium parameter $R_{L}$, may be used to represent the Langmuir isotherm model, as illustrated in Eq. 17:

17 $$ R_{L} = \frac{1}{{1 + K_{L} C_{0} }} $$

$$C_{0} $$ is the initial metal ion concentration ($$mg.L^{ - 1}$$), if $$R_{L}$$ is zero, the isotherm is irreversible, if it is unfavorable ($$R_{L} > 1)$$, it is linear if $$R_{L} = 1$$, it is favorable ($$0 < R_{L} <$$ 1). The $$R_{L}$$ values in this study range from 0.003–0.348 (0 $$< R_{L} <$$ 1), as shown in Table 6, which supports the successful adsorption of Mo(VI) onto the adsorbent^67^.

#### Adsorption kinetics models

Many kinetic models may be employed to represent the mechanism of Mo(VI) adsorption onto TEA-GMA-g-PAN. Numerous kinetic models were used to assess the experimental data after studies on adsorption rate were conducted to evaluate the governing mechanisms of the adsorption process, such as diffusion control, chemical reaction, and mass transfer. Three kinetics models, namely the pseudo-first-order (Eq. 18)^88^, pseudo-second-order (Eq. 19)^89^, and intra-particle diffusion (Weber–Morris) models (Eq. 20), were used to assess the kinetics data collected from batch tests^90^.

18 $$ q_{t} = q_{e,cal} \left( {1 - e^{{ - k_{1} t}} } \right) $$

19 $$ q_{t} = \frac{{k_{2} q_{e,cal}^{2} t}}{{1 + k_{2} q_{e,cal} t}} $$

20 $$ q_{t} = K_{i} t^{0.5} + C $$

where $q_{e} , q_{e,cal}$, and $q_{t}$ are the experimental adsorption capacity ($mg.g^{ - 1}$), calculated adsorption capacity ($mg.g^{ - 1}$) and adsorption capacity ($mg.g^{ - 1}$) at time t (min), respectively. $K_{1}$ ($min.^{ - 1}$) relates to the rate constant of the pseudo-first-order and $K_{2} \left( {g.mg^{ - 1} .{\text{min}}.^{ - 1} } \right)$ relates to the rate constant of the pseudo-second-order and the nonlinear plot of the curve $q_{t}$ versus time (min) was used to calculate the values of the rate constant ($K_{1}$) and $K_{2}$. where, $K_{i} \left( {mg.g^{ - 1} .min.^{ - 0.5} } \right)$ is the intraparticle diffusion rate constant of stage I, C the intercept of stage I, gives an idea about the thickness of the boundary layer.

**Figure 11:**
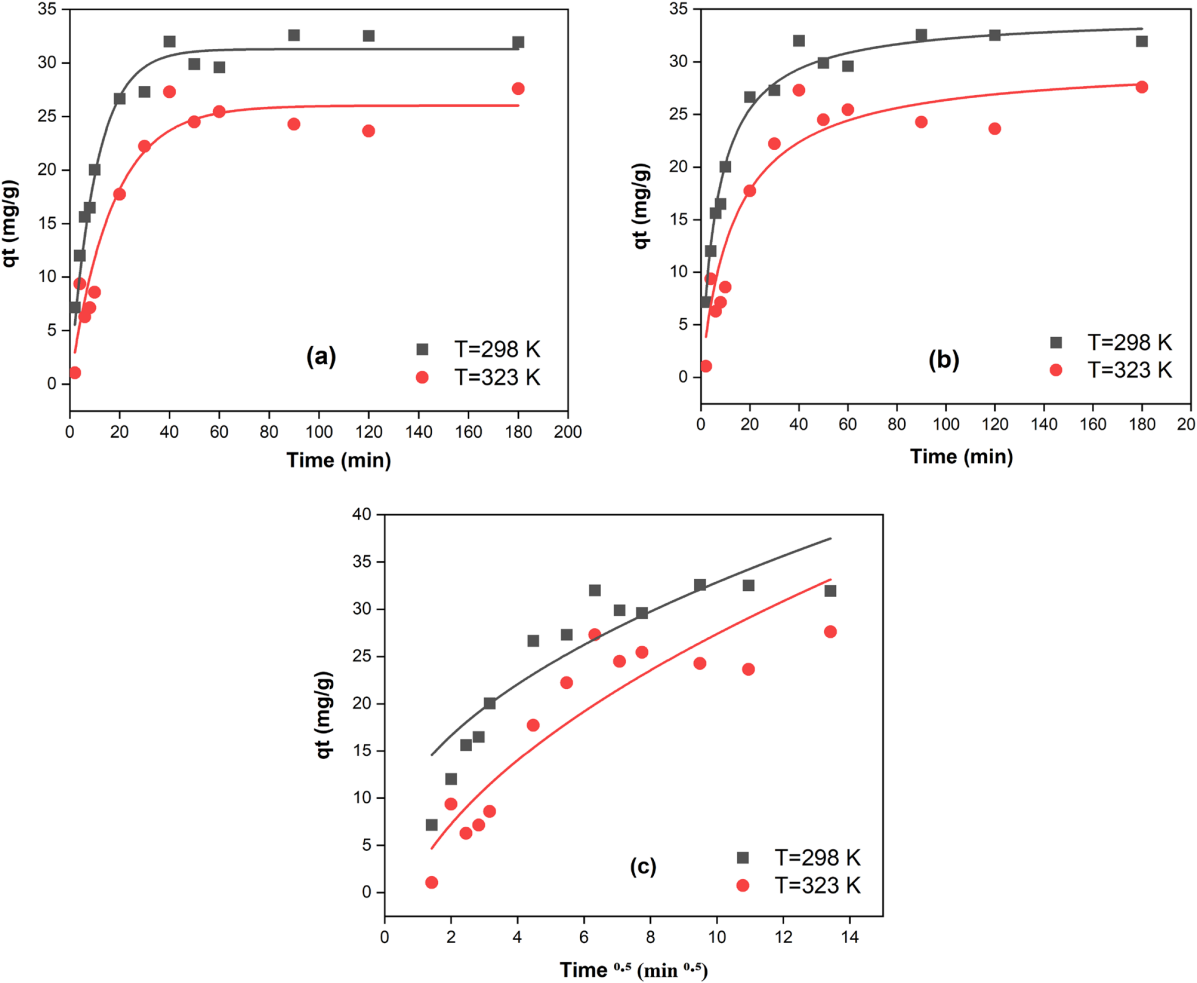
Effect of contact time at different temperatures. (**a**) pseudo first-order, (**b**) pseudo second-order and (**c**) Weber–Morris plots for the adsorption of Mo (VI).

Figure 11 depicts the kinetic modeling of Mo(VI) adsorption on Amine-GMA-g-PAN. Additionally, Table 7 provides an overview of the determination coefficient values ($R^{2}$) and adsorption kinetic constants for three kinetic models. The pseudo-second-order kinetics model's determination coefficient for the adsorption of Mo(VI) by Amine-GMA-g-PAN is hugely near to unity. Additionally, the estimated value of the pseudo-second-order kinetics model and the observed value of adsorption capacity are pretty similar. These data suggest that the pseudo-second-order kinetics model, rather than the pseudo-first-order model and the Weber–Morris model, may more accurately represent the adsorption process. This discovery indicates that the adsorption of Mo(VI) onto Amine-GMA-g-PAN is a chemical coordination process.

**Table 7:** Kinetic parameters and correlation coefficients of pseudo-first-order, pseudo-second-order and Weber–Morris models for the adsorption of Mo (VI).

Temp. K	Pseudo-first-order parameter			Pseudo-second-order parameter			$q_{{{\text{exp}}}} \left( \frac{mg}{g} \right)$	Weber–Morris		
	$k_{1}$ $\left( {{\text{min}}.^{ - 1} } \right)$	$q_{e,cal} \left( {mg/g} \right)$	$R^{2}$	$k_{2} \left( {\frac{g}{mg.min.}} \right)$	$q_{e,cal} \left( {mg/g} \right)$	$R^{2}$		$K_{i}$	$C$	$R^{2}$
298	0.105	31.116	0.976	0.0042	34.426	0.999	32.59	9.27	3.53	0.975
323	0.056	26.19	0.943	0.0025	29.969	0.998	27.61	11.51	− 9.02	0.972

#### Thermodynamic parameters

**Figure 12:**
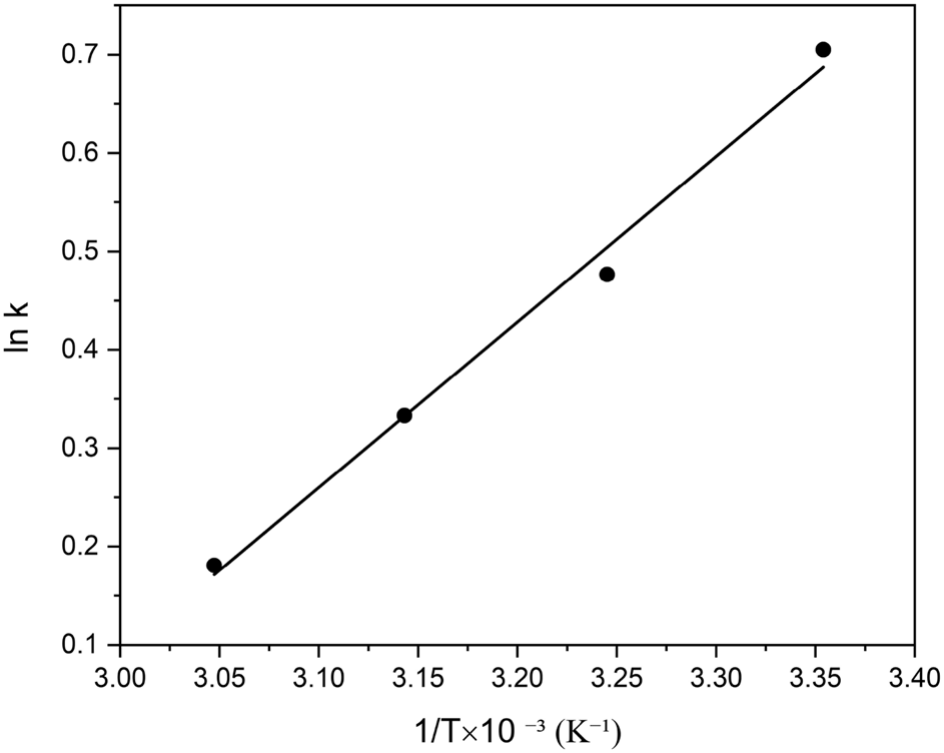
Linear plot of ln k versus 1/T for the adsorption of Mo on adsorbent.

Gibbs free energy ($\Delta G^\circ$), enthalpy ($\Delta H^\circ$), and entropy ($\Delta S^\circ$) are the thermodynamic quantities that serve as the actual indications for the practical application of Mo(VI) adsorption process onto nanofiber. What procedure will happen spontaneously is often governed by the values of these factors. Equations (21–23) are used to compute Gibb's free energy ($\Delta G^\circ$)^91,92^:

21 $$ \Delta G^\circ = \Delta H^\circ - T\Delta S^\circ = - RTlnk $$

22 $$ lnk = \ln \left( {\frac{{q_{e} }}{{C_{e} }}} \right) $$

23 $$ \ln k = \frac{ - \Delta G^\circ }{{RT}} = - \frac{\Delta H^\circ }{{RT}} + \frac{\Delta S^\circ }{R} $$

where *k* is the distribution coefficient at each temperature, *T* is the solution temperature (*K*); *R* is the universal gas constant (8.314 $J.mol^{ - 1} .K^{ - 1}$ ); the values of ΔH and ΔS were determined from the slope and intercept value of the linear plot of ln *k* versus 1/T (see in Fig. 12). At 298 K, 308 K, 318 K, and 328 K, equilibrium experiments were conducted with a Mo(VI) solution containing 50 mg/L and the acquired thermodynamic parameters. These experiments' results are presented in Fig. 12 and Table 8. The advantageous and random nature of the adsorption process is demonstrated by the negative numbers of the Gibbs free energy, Additionally, raising the temperature causes the reaction's degree of the spontaneity to decrease. The exothermic nature of the adsorption process is indicated by the negative number of $\Delta H$. Additionally, the drop in unpredictability during the adsorption process, which is connected to the formation of a steady structure upon attachment of the Mo(VI) ions onto the binding sites of the adsorbent, can be used to explain the negative value of entropy^67^.

**Table 8:** Thermodynamic parameters for Mo adsorption on adsorbent.

T (K)	$\Delta G^\circ \left( {J.mol^{ - 1} } \right)$	$\Delta H^\circ \left( {J.mol^{ - 1} } \right)$	$\Delta S^{ \circ } \left( {J.mol^{ - 1} .K^{ - 1} } \right)$
298	− 1747.61	− 13.99	− 41.21
308	− 1221.08		
318	− 881.21		
328	− 493.33		

### Desorption stage

**Figure 13:**
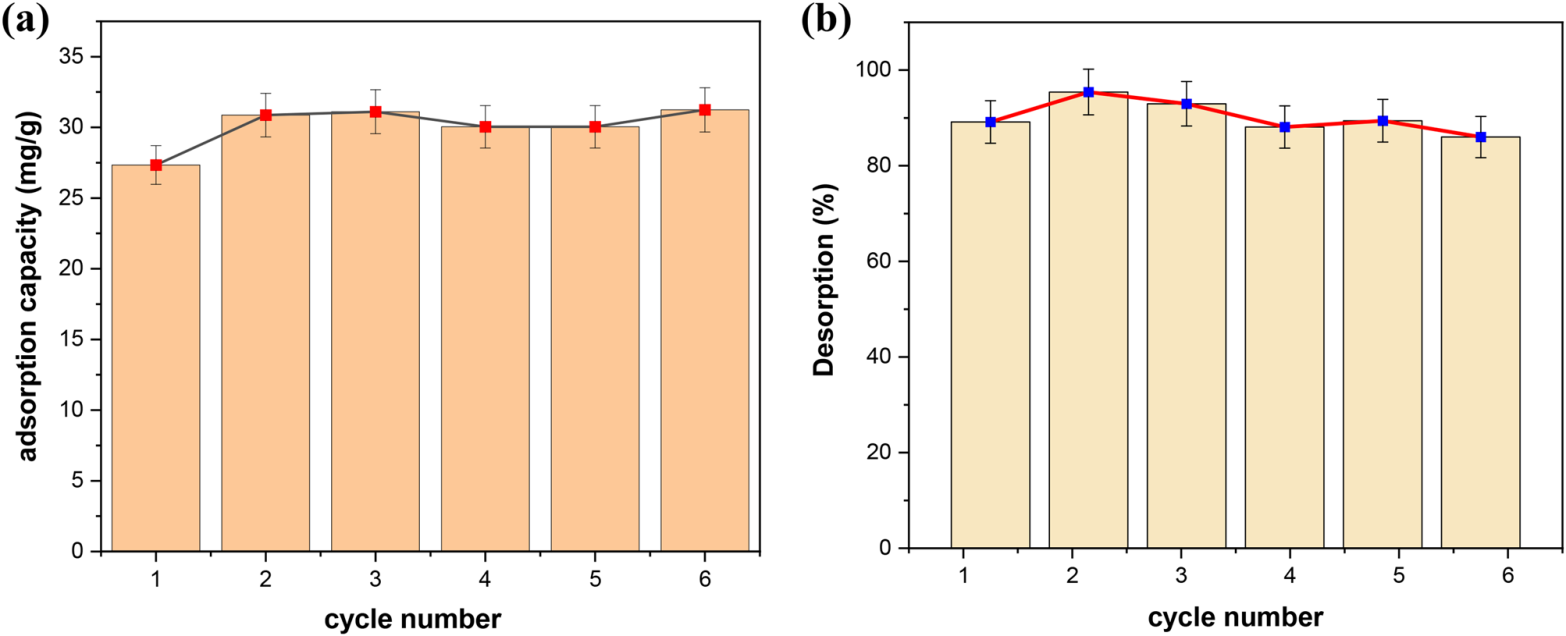
Adsorption–desorption performances of Mo on the nanofiber upon six recycles.

A crucial factor in assessing an adsorbent in real uses is how well it recycles and regenerates. In the cycling test, ${\text{HCl}}$ (0.5 M) solution for 4 h was used to regenerate Mo(VI)-loaded adsorbent after the adsorption equilibrium. According to Fig. 13, the adsorption capabilities of the adsorbent for Mo(VI) are nearly constant after six rounds of adsorption–desorption, while the desorption efficacy fell by 9.8%. (Fig. 13b). After six rounds, it appears that the adsorbent's high adsorption capability can still be kept.

### Selectivity stage

Molybdenite concentrate is roasted at the Sarcheshmeh Copper Co. in Kerman, Iran, to create molybdenum as a by-product. In this study, molybdenum and other metals (Cu, Fe, Al, and Ca) from the residual leach solution were focused on for adsorption. The goal of this study was to conduct batch tests to determine the selectivity at which molybdenum could be calculated.

The feed solution was the actual leach solution, which contained molybdenum ore and 1:1 ratios of hydrochloric acid (12 mol/L) and nitric acid (14 mol/L). The pH of the solution was adjusted to be between 1 and 13, and then 0.1 g of the synthesized adsorbent was added. The residual Mo, Fe, Ca, Al, and Cu content in the aqueous solution was then measured by ICP analysis after the flask had been shaken at 298 K and 200 rpm for four hours. Table 9 displays the concentrations of various elements in the mixture.

**Table 9:** The concentration of elements in molybdenite ore leach solution at different pH in the adsorption process.

Element	$C_{i} \left( {ppm} \right)$	$C_{e} \left( {ppm} \right)$	$k_{d}$	$\alpha$
pH = 1				
Al	26.33	15.21	358.38	1.44
Ca	16.96	16.64	9.42	54.76
Fe	77.88	77.63	1.57	327.05
Mo	404.9	197.2	516.29	1
Cu	41.71	41.35	4.26	120.97
pH = 3				
Al	26.72	10.63	591.84	1.34
Ca	17.76	17.16	13.67	58.15
Fe	20.94	18.64	48.24	16.47
Mo	418.9	138.1	795.04	1
Cu	42.15	40.69	14.03	56.66
pH = 5				
Al	24.72	12.17	300.94	0.54
Ca	16.87	16.7	2.97	55.24
Fe	19.3	8.01	411.32	0.39
Mo	378.4	242.2	164.11	1
Cu	34.85	33	16.36	10.03
pH = 7				
Al	25.89	19.43	95.72	0.81
Ca	13.43	13.26	3.69	21.01
Fe	0.83	0.24	707.77	0.11
Mo	486.9	383.6	77.53	1
Cu	2.04	1.21	197.49	0.39
pH = 9				
Al	22.18	22.04	1.88	6.83
Ca	14.43	12.05	58.49	0.22
Fe	0.34	0.09	822.63	0.01
Mo	447.1	428.5	12.85	1
Cu	1.8	0.58	622.93	0.02
pH = 11				
Al	27.63	27.31	3.45	0.33
Ca	4.95	4.94	0.59	1.96
Fe	0.32	0.28	42.09	0.027
Mo	479.4	477.5	1.17	1
Cu	1.8	0.54	687.62	0.002
pH = 13				
Al	32.93	32.83	0.87	0.27
Ca	3.78	3.75	2.31	0.11
Fe	0.58	0.56	10.31	0.023
Mo	469.7	469.3	0.24	1
Cu	2.22	1.34	189.62	0.001

**Figure 14:**
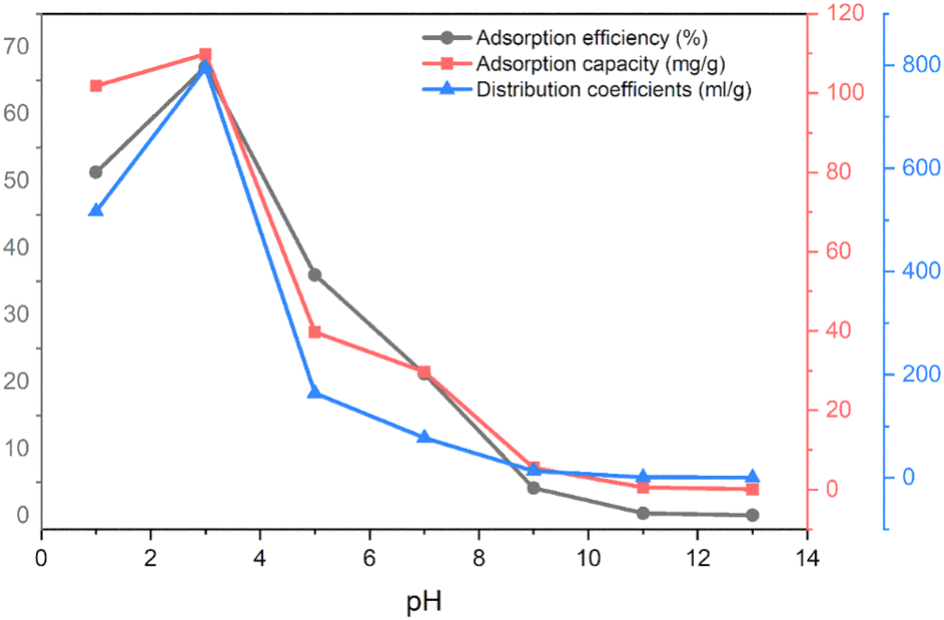
Effect of pH on adsorption efficiency, adsorption capacity, and distribution coefficient of molybdenum in molybdenite ore leach solution.

According to Table 9 and Fig. 14, the distribution coefficient, adsorption efficiency, and adsorption capacity are the highest at pH = 3, and the synthesized adsorbent selectively adsorbs molybdenum, but molybdenum adsorption decreases at pH > 3. Practically, at a pH higher than 8, due to the change in the surface charge of the adsorbent, the adsorption of molybdenum reaches almost zero. At a pH lower than 3 (acidic medium), because the molybdenum is converted into $Mo^{3 + }$ form in aqueous solution, the adsorption of molybdenum also decreases.

## Conclusion

In the current work, the adsorbent was generated by gamma-irradiation through copolymerization of glycidyl methacrylate (GMA) onto the surface of electrospun PAN nanofiber, followed by chemical modification with triethylamine for molybdenum adsorption from aqueous solutions. Modification with amine opens the ring of epoxy glycidyl methacrylate and converts tertiary amine into quaternary ammonium, and on the other hand, Sodium molybdate dihydrate is hydrolyzed in water and converted into molybdate ($MoO_{4}^{2 - }$), and because molybdate contains two negative charges, it is adsorbed by quaternary ammonium. The GMA concentration, pH, temperature, initial concentration, contact time, adsorbent mass, irradiation dosage, and amine concentration all impacted on the adsorption capacity of Mo(VI). The results showed that using the produced TEA-GMA-g-PAN as an efficient nanofiber adsorbent for the selective molybdenum adsorption from an aqueous solution was possible.

### Supplementary Information

Supplementary Tables.

## Data Availability

The datasets used and/or analysed during the current study available from the corresponding author on reseanable request.
