# Supplementary material for: Targeted elimination of molybdenum ions from a leaching solution with the ability of radiated grafting GMA-PAN nanofibers
Source: Sci Rep. 2024 Jan 2;14:252. doi: 10.1038/s41598-023-50608-0 (PMC10762185; doi:10.1038/s41598-023-50608-0)
Supplement: Supplementary file 1 — Supplementary Tables. [file 41598_2023_50608_MOESM1_ESM.docx]

**Table A.** Independent variables and result for the degree of grafting and adsorption capacity of Mo(VI) by CCD.

| Run | GMA% | Dose (kGy) | Amine% | DG%  (exp.) | DG%  (model) | Error% | Adsorption capacity(mg/g)  (exp.) | Adsorption capacity(mg/g)  (model) | Error  % |
| --- | --- | --- | --- | --- | --- | --- | --- | --- | --- |
| 1 | 25 | 40 | 40 | 724.538 | 717.96 | 0.91 | 17.506 | 17.88 | 2.15 |
| 2 | 25 | 20 | 80 | 690.934 | 692.79 | 0.27 | 14.937 | 15.31 | 2.51 |
| 3 | 15 | 20 | 40 | 439.655 | 433.02 | 1.51 | 17.393 | 17.76 | 2.10 |
| 4 | 30 | 30 | 60 | 858.23 | 856.20 | 0.24 | 17.328 | 16.97 | 2.02 |
| 5 | 20 | 50 | 60 | 612.73 | 625.92 | 2.15 | 18.899 | 19.24 | 1.78 |
| 6 | 25 | 20 | 40 | 660.27 | 662.42 | 0.33 | 18.451 | 18.77 | 1.74 |
| 7 | 20 | 30 | 60 | 553.729 | 560.65 | 1.25 | 19.899 | 19.79 | 0.53 |
| 8 | 15 | 20 | 80 | 485.426 | 480.78 | 0.96 | 16.131 | 15.76 | 2.29 |
| 9 | 20 | 10 | 60 | 585.765 | 583.79 | 0.34 | 18.296 | 17.95 | 1.86 |
| 10 | 20 | 30 | 60 | 534.511 | 560.65 | 4.90 | 19.690 | 19.79 | 0.52 |
| 11 | 15 | 40 | 80 | 480.729 | 467.37 | 2.78 | 18.243 | 17.93 | 1.71 |
| 12 | 20 | 30 | 60 | 582.5 | 560.65 | 3.75 | 19.792 | 19.95 | 0.69 |
| 13 | 20 | 30 | 20 | 499.07 | 505.54 | 1.29 | 15.342 | 14.99 | 2.24 |
| 14 | 15 | 40 | 40 | 426.991 | 413.91 | 3.06 | 21.885 | 21.52 | 1.68 |
| 15 | 10 | 30 | 60 | 326.881 | 340.13 | 4.05 | 20.716 | 21.06 | 1.67 |
| 16 | 20 | 30 | 100 | 584.641 | 589.39 | 0.81 | 7.609 | 7.95 | 4.48 |
| 17 | 25 | 40 | 80 | 758.64 | 754.05 | 0.61 | 13.190 | 12.83 | 2.72 |

**Table B.** Analysis of variance (ANOVA) in the first experiment design for response surface quadratic model for adsorption capacity (Eq. (10)).

| **Source** | **Sum of squares** | **df** | **Mean square** | **F-value** | **P-value** |
| --- | --- | --- | --- | --- | --- |
| **Model** | 176.94 | 9 | 19.66 | 78.88 | <0.0001 |
| **A-GMA** | 16.69 | 1 | 16.69 | 66.97 | <0.0001 |
| **B-Dose** | 1.64 | 1 | 1.64 | 6.57 | 0.0374 |
| **C-Amine** | 49.70 | 1 | 49.70 | 199.40 | <0.0001 |
| **AB** | 10.80 | 1 | 10.80 | 43.34 | 0.0003 |
| **AC** | 1.07 | 1 | 1.07 | 4.29 | 0.0770 |
| **BC** | 1.26 | 1 | 1.26 | 5.07 | 0.0590 |
| $\boldsymbol{A}^{\boldsymbol{2}}$ | 0.7224 | 1 | 0.7224 | 2.90 | 0.1325 |
| $\boldsymbol{B}^{\boldsymbol{2}}$ | 1.73 | 1 | 1.73 | 6.95 | 0.0336 |
| $\boldsymbol{C}^{\boldsymbol{2}}$ | 83.77 | 1 | 83.77 | 336.10 | <0.0001 |
| **Residual** | 1.74 | 7 | 0.2492 |  |  |
| **Lack of fit** | 1.72 | 5 | 0.3446 | 31.47 | 0.0311 |
| **Pure error** | 0.0219 | 2 | 0.0109 |  |  |
| **Cor total** | 178.68 | 16 |  |  |  |

**Table C.** Standard deviation and $R^{2}$ in the first experiment design for degree of grafting (Eq. (9)) and adsorption capacity (Eq. (10)).

| **Degree of Grafting (Eq. (9)** | **Std. Dev.** | 17.40 | $\boldsymbol{R}^{\boldsymbol{2}}$ | 0.9926 |
| --- | --- | --- | --- | --- |
|  | **Mean** | 576.78 | **Adjusted** $\boldsymbol{R}^{\boldsymbol{2}}$ | 0.9830 |
|  | **C.V %** | 3.02 | **Predicted** $\boldsymbol{R}^{\boldsymbol{2}}$ | 0.9644 |
|  |  |  | **Adequate precision** | 38.677 |
| **Adsorption Capacity (Eq. (10)** | **Std. Dev.** | 0.4992 | $\boldsymbol{R}^{\boldsymbol{2}}$ | 0.9902 |
|  | **Mean** | 17.37 | **Adjusted** $\boldsymbol{R}^{\boldsymbol{2}}$ | 0.9777 |
|  | **C.V %** | 2.87 | **Predicted** $\boldsymbol{R}^{\boldsymbol{2}}$ | 0.9201 |
|  |  |  | **Adequate precision** | 35.4383 |

**Table D.** Independent variables and result for adsorption capacity of Mo(VI) by CCD in the second experiment design.

| Run | pH | Initial concentration (mg/L) | Adsorbent mass (g) | Adsorption capacity(mg/g)  (exp.) | Adsorption capacity(mg/g)  (model) | Error  % |
| --- | --- | --- | --- | --- | --- | --- |
| 1 | 5 | 20 | 0.15 | 5.89 | 5.64 | 4.16 |
| 2 | 6.5 | 40 | 0.2 | 15.73 | 15.98 | 1.59 |
| 3 | 5 | 60 | 0.15 | 25.72 | 26.42 | 2.75 |
| 4 | 5 | 60 | 0.05 | 39.93 | 39.62 | 0.76 |
| 5 | 5 | 60 | 0.15 | 26.61 | 26.42 | 0.7 |
| 6 | 6.5 | 80 | 0.1 | 30.82 | 31.13 | 1.01 |
| 7 | 3.5 | 80 | 0.2 | 22.77 | 22.58 | 0.87 |
| 8 | 5 | 60 | 0.15 | 26.94 | 26.43 | 1.92 |
| 9 | 5 | 60 | 0.25 | 16.10 | 16.71 | 1.87 |
| 10 | 2 | 60 | 0.15 | 27.34 | 27.55 | 0.74 |
| 11 | 6.5 | 80 | 0.2 | 21.42 | 21.06 | 1.69 |
| 12 | 5 | 100 | 0.15 | 22.16 | 22.41 | 1.11 |
| 13 | 8 | 60 | 0.15 | 25.41 | 25.21 | 0.79 |
| 14 | 3.5 | 80 | 0.1 | 37.25 | 36.99 | 0.69 |
| 15 | 6.5 | 40 | 0.1 | 24.29 | 24.48 | 0.79 |
| 16 | 3.5 | 40 | 0.2 | 12.77 | 12.45 | 2.47 |
| 17 | 3.5 | 40 | 0.1 | 24.95 | 25.30 | 1.43 |

**Table E.** Analysis of variance (ANOVA) for response surface quadratic model for adsorption capacity in the second experiment design.

| **Source** | **Sum of squares** | **df** | **Mean square** | **F-value** | **P-value** |
| --- | --- | --- | --- | --- | --- |
| **Model** | 1098.6 | 9 | 122.01 | 461.19 | <0.0001 |
| **A-pH** | 5.47 | 1 | 5.47 | 20.67 | 0.0026 |
| **B-initial co.** | 281.10 | 1 | 281.10 | 1062.56 | <0.0001 |
| **C-adsorbent mass** | 525.18 | 1 | 525.18 | 1985.17 | <0.0001 |
| **AB** | 12.74 | 1 | 12.74 | 48.17 | 0.0002 |
| **AC** | 9.45 | 1 | 9.45 | 35.72 | 0.0006 |
| **BC** | 1.24 | 1 | 1.24 | 4.67 | 0.0675 |
| $\boldsymbol{A}^{\boldsymbol{2}}$ | 0.0027 | 1 | 0.0027 | 0.0101 | 0.9226 |
| $\boldsymbol{B}^{\boldsymbol{2}}$ | 186.02 | 1 | 186.02 | 703.16 | <0.0001 |
| $\boldsymbol{C}^{\boldsymbol{2}}$ | 3.67 | 1 | 3.67 | 13.88 | 0.0074 |
| **Residual** | 1.85 | 7 | 0.2646 |  |  |
| **Lack of fit** | 1.05 | 5 | 0.2097 | 0.5223 | 0.7587 |
| **Pure error** | 0.8032 | 2 | 0.4016 |  |  |
| **Cor total** | 1099.91 | 16 |  |  |  |

**Table F.** Standard deviation and $R^{2}$ for adsorption capacity in the second experiment design.

| **Std. Dev.** | 0.5143 | $\boldsymbol{R}^{\boldsymbol{2}}$ | 0.9983 |
| --- | --- | --- | --- |
| **Mean** | 23.91 | **Adjusted** $\boldsymbol{R}^{\boldsymbol{2}}$ | 0.9962 |
| **C.V %** | 2.15 | **Predicted** $\boldsymbol{R}^{\boldsymbol{2}}$ | 0.9907 |
|  |  | **Adequate precision** | 86.14 |
